# Heat Shock Response in Yeast Involves Changes in Both Transcription Rates and mRNA Stabilities

**DOI:** 10.1371/journal.pone.0017272

**Published:** 2011-02-25

**Authors:** Laia Castells-Roca, José García-Martínez, Joaquín Moreno, Enrique Herrero, Gemma Bellí, José E. Pérez-Ortín

**Affiliations:** 1 Departament de Ciències Mèdiques Bàsiques and IRBLleida, Universitat de Lleida, Lleida, Catalunya, Spain; 2 Departamento de Bioquímica y Biología Molecular, Facultad de Ciencias Biológicas, Universitat de València, Burjassot, Valencia, Spain; 3 Sección de Chips de DNA-Servei Central de Suport a la Investigació Experimental, Universitat de València, Burjassot, Valencia, Spain; University College London, United Kingdom

## Abstract

We have analyzed the heat stress response in the yeast *Saccharomyces cerevisiae* by determining mRNA levels and transcription rates for the whole transcriptome after a shift from 25°C to 37°C. Using an established mathematical algorithm, theoretical mRNA decay rates have also been calculated from the experimental data. We have verified the mathematical predictions for selected genes by determining their mRNA decay rates at different times during heat stress response using the regulatable *tetO* promoter. This study indicates that the yeast response to heat shock is not only due to changes in transcription rates, but also to changes in the mRNA stabilities. mRNA stability is affected in 62% of the yeast genes and it is particularly important in shaping the mRNA profile of the genes belonging to the environmental stress response. In most cases, changes in transcription rates and mRNA stabilities are homodirectional for both parameters, although some interesting cases of antagonist behavior are found. The statistical analysis of gene targets and sequence motifs within the clusters of genes with similar behaviors shows that both transcriptional and post-transcriptional regulons apparently contribute to the general heat stress response by means of transcriptional factors and RNA binding proteins.

## Introduction

Cells respond to a variety of environmental stresses by reprogramming the expression of specific sets of genes which depend on the particular stress. In yeast (*Saccharomyces cerevisiae*), this response has been extensively studied at the genome level for a number of stresses [1], [2]. Most genomic studies on stress responses involve the determination of mRNA amounts (abbreviated as RA herein); however for each gene, RA depends on the equilibrium between the transcription rate (TR) and the decay rate of each particular mRNA [3]. We have shown that in the dynamic situation after applying oxidative stress to yeast cells, the transitory changes in the decay rate of different mRNAs are as relevant as the transcriptional shifts in modulating the RA changes that take place during adaptation to stress [4]. This relevance can be extended to other stresses and organisms [5]–[7].

The heat shock response in yeast is mediated by the transcription factors (TF) Hsf1 and Msn2/Msn4, and involves the upregulation of a number of genes for heat-shock proteins (Hsps) that participate in protein folding, trafficking and maturation, and also the genes for the protein degradation machinery. This is paralleled by a downregulation of the genes for ribosome biogenesis [8]. Some Hsp genes are specific for one of the above transcription factors, while others are governed by both Hsf1 and Msn2/4 through the respective HSE and STRE promoter motifs. Heat shock factor Hsf1 is an essential protein in *S. cerevisiae*, which participates in both the heat shock response and the adaptation to oxidative stress and glucose starvation [9], [10]. The partially redundant zinc-finger transcription factors Msn2 and Msn4 mediate the response to a variety of cellular and environmental stresses, including heat shock (known as the Environmental Stress Response, reviewed in [11]). During adaptation to heat shock, both factors seem to play differential roles: Hsf1 is essential for recovery from a brief exposure to extreme temperature, while Msn2/4 is required for long-term survival at high temperatures [12]. Partial overlapping has been described between the heat shock response and the unfolded protein response, which is induced by misfolded proteins at the endoplasmic reticulum and mediated by the Hac1 TF [13]. Both types of stress share a number of transcription targets.

The Hsf1-mediated response of yeast cells subjected to heat shock has been studied at the systemic level through a combination of microarray analyses of RA and promoter occupancy determination by chromatin immunoprecipitation [14]. Heat shock mainly induces the binding of Hsf1 to the promoters of its target genes, which represent nearly 3% of the yeast genome. In addition to the genes for protein folding, degradation and trafficking, Hsf1 targets are also implicated in biological functions such as maintenance of cell integrity, molecular transport, cell signaling and transcription [9]. However, the above studies do not discriminate between the relative roles of TR and mRNA decay changes as determinants of the dynamics of mRNA levels during the heat shock response. The genomic run-on (GRO) methodology allows the quantification of the TR and RA parameters for each particular gene on a genomic scale, and subsequently allows the determination of the relative values of mRNA decay rate (*k_D_*) for each gene [3], [15]. This technique has been employed to determine changes in mRNA stability in the *S. cerevisiae* transcriptome during a nutritional shift from glucose to galactose [16], and during oxidative stress [4]. The latter study demonstrated that changes in mRNA stability modulate the stress response together with the TR dynamics for particular gene subsets [4]. Following a different approach, other authors have demonstrated that alterations in the mRNA decay rate determine the speed and relaxation properties of the oxidative stress response for specific mRNAs [17]. Modulation of mRNA stability also influences the response of yeast cells during osmotic stress, as revealed by studies using transcription inhibitors [18] or GRO [6] for mRNA decay determinations. In the present study, we find that the main regulatory response upon heat shock occurs at the TR level, although this is modulated by adjusting the mRNA stability of specific sets of genes. In most cases, such changes in mRNA stability are homodirectional to those in the transcription rate and are putatively conducted by known or unknown RNA binding proteins (RBP).

## Results

### General Cell Response to Moderate Heat Stress

**Figure 1:**
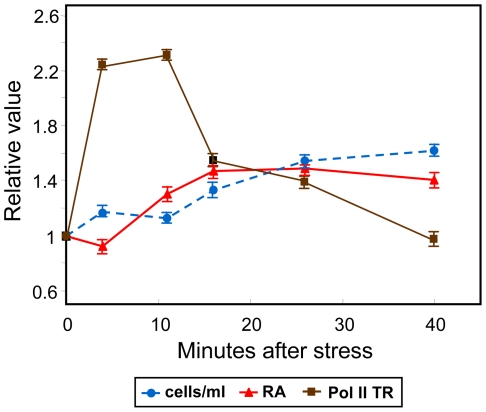
Time course of the heat shock experiment. At time 0, cells growing exponentially at 25°C were shifted to 37°C. At the indicated times, aliquots were taken to measure cell concentration, total mRNA per cell (RA) and Pol II transcription rate (TR) per cell (see “Materials and Methods”). The parameters were referred to their respective time 0 values. *Bars*: standard deviation (n = 3).

It has been described that *S. cerevisiae* cells subjected to intense heat shock treatment show a transient growth arrest at the G1 stage of the cell cycle. After a time, cells spontaneously recover and resume cell cycle progression, even under high temperature conditions [19]. To minimize undesirable side effects on the cell cycle, which could disturb the direct effects of heat shock on general transcription and mRNA stability, we employed mild stress conditions that minimally affected exponential growth (cells continue growing, see Fig. 1), but still induced the expression of the genes taking part in the heat shock response. For this purpose, we applied heat stress by shifting exponentially growing cells from 25°C to 37°C, and studied the general transcriptional response of cells under such stress. Other studies involving heat shock employed a broader temperature change.

In this study, the total amount of poly(A) mRNA per cell increased suddenly from 5 min to 20 min after stress and, afterwards maintaining the level achieved (Fig. 1). Whole RNA polymerase II (Pol II) transcription increased initially with regard to time 0, an effect which was also observed after a carbon source shift and upon oxidative stress [4], [16]. Part of this effect was due to the unspecific increase of enzyme activity caused by temperature rise (see M&M). Then Pol II transcription decreased to reach levels about those at time 0. Thus, comparison of the kinetics of the mRNA levels and Pol II-dependent transcription under heat shock conditions suggested general mRNA stabilization after a quick transient destabilization.

### Effect of Moderate Heat Shock on mRNA Levels and Transcription Rates on Individual Genes

**Figure 2:**
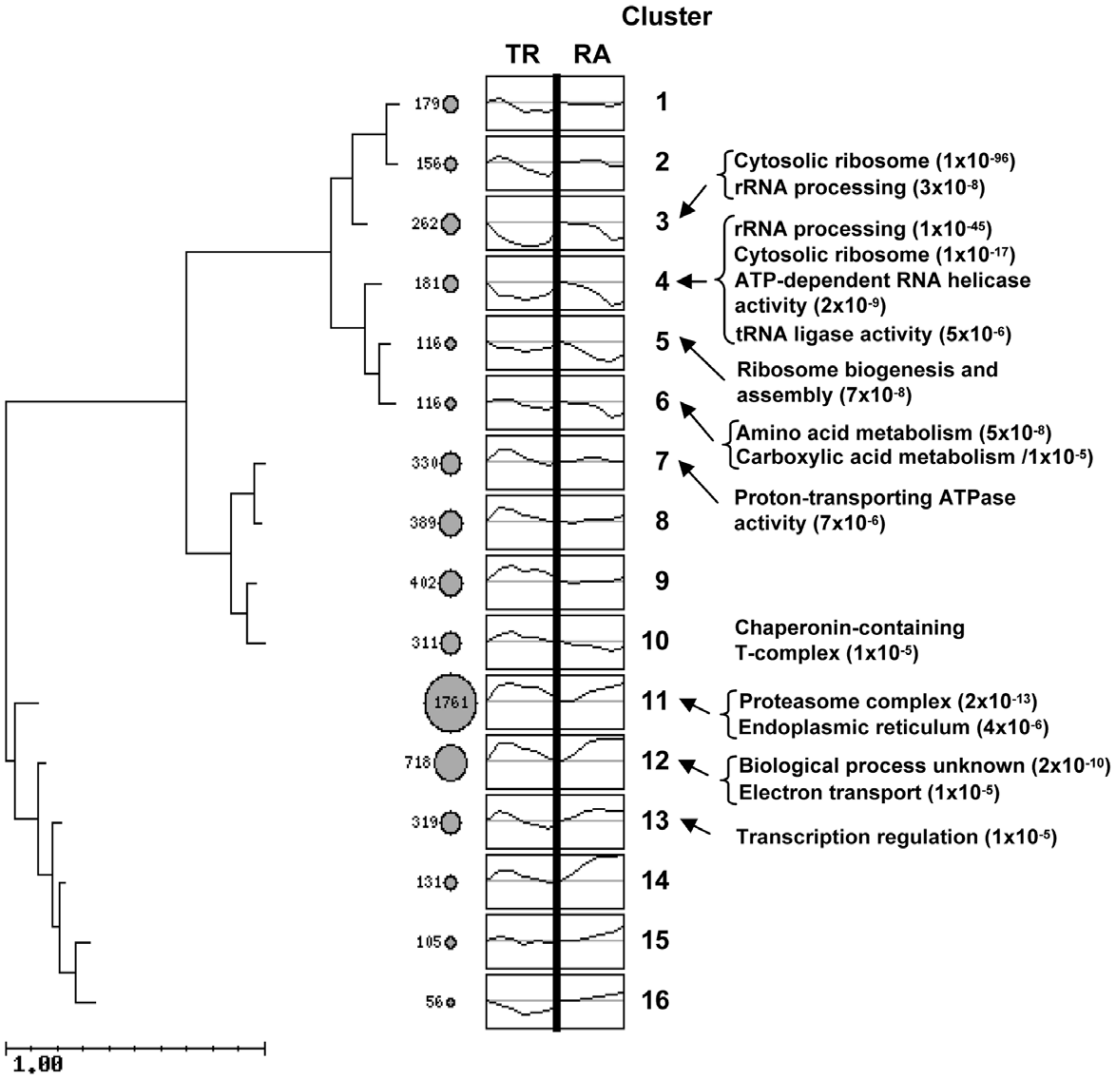
Clustering of TR and RA data. Time course profiles for both parameters were considered for clustering. Both dataset series are normalized to time 0 value to allow a comparison between the TR and RA data. The discontinuation between the last TR point and the time 0 RA value has no real meaning and is represented as a *vertical black bar*. For each cluster in the tree, the number of genes and data profiles are indicated. Ordinates are on a log scale, and the *horizontal line* in each graph marks the time 0 level. The most significant GO categories (*p* value ≤10^−5^) are shown. The individual data for each gene and the list of genes in each cluster can be seen in Tables S1 and S2, respectively. The *scale bar* on the *lower left side* reflects the distances between the cluster profiles.

By following the GRO experimental procedures [16], we have determined the TR and RA values for 5532 yeast genes during the *S. cerevisiae* cell response to moderate heat shock. The signals obtained for both parameters were normalized by genomic DNA hybridization to fully compare the values obtained for individual genes. These values are listed in Table S1. Both the TR and RA data were used to perform the clustering analyses. Values relative to t_0_ were used to avoid differences in scale for these two datasets. Therefore, TR and RA at t_0_ take a value of 0 in the log scale. The ten-point profiles obtained reflect the variation of TR (first 5 points) and its effect on RA (last 5 points, Fig. 2). As in a previous work [4], most of the mRNAs in our experiment are not under steady-state conditions (RA is not constant) and, therefore, RA profiles depend on both TR and mRNA stability according to kinetic laws [3].

Table S2 lists the genes included in all 16 resulting clusters. The cluster-profile tree (Fig. 2) shows a main branch (clusters 1–10), characterized by decreasing or stable RA levels, which includes 2442 genes (45% of the total number), and which is subdivided into two sub-branches. The first (clusters 1–6) includes genes that display a decrease in the TR levels during the time course, followed in most cases by a drop in RA. Therefore, they correspond to repressed categories (Table S2). The genes in this sub-branch (19% of the total) are regularly distributed among clusters, where clusters 3 and 4 (enriched in the ‘Cytosolic ribosome’ and ‘rRNA processing’ Gene Ontology, GO, categories) contain the largest number of genes. Cluster 6 in this sub-branch is enriched in amino acid metabolism genes, which also drop in both TR and RA. The most interesting behavior is seen in clusters 3–5, which show an immediate TR decrease, whereas RA only changes significantly at later times. The second sub-branch in the upper part of the tree (clusters 7–10, 26% of the total genes) displays a different behavior. In this case, TR is temporarily and moderately upregulated, whereas RA remains unchanged in most cases (clusters 7–9), or even decreases (cluster 10), suggesting strong destabilization effects.

**Figure 3:**
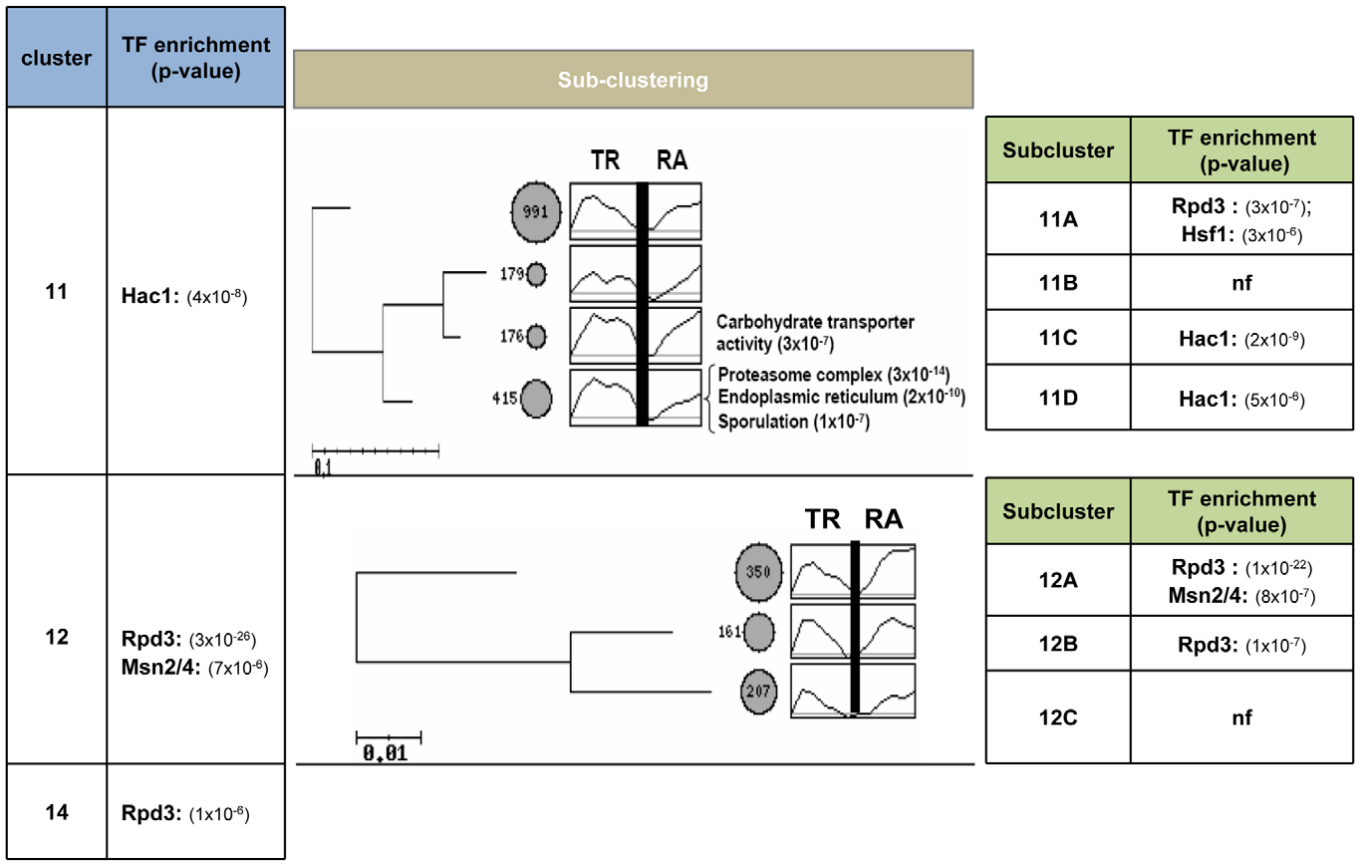
Sub-partition of genes in clusters 11 and 12 and Transcription Factor enrichment analysis. The genes in clusters 11 and 12 of Figure 2 (listed in Table S2) were partitioned into subclusters using the same criteria as for the general clustering analysis. See the legend of Figure 2 for details of the representation. The most significant GO categories in each resulting subcluster (*p* value≤10^−5^) are shown on the right. Transcription factor targets enrichment in each cluster (left) or subcluster (right) is shown with its *p*-value. nf: not significant categories found.

The lower part of the tree (clusters 11–16) may be considered as a whole because of the short branches connecting the clusters. In all cases, RA is upregulated upon heat shock which, in most cases, is paralleled by a more or less sustained increase of TR. The exceptions are clusters 15 and 16, where TR remains constant (cluster 15) or decreases (cluster 16), while RA increases in a sustained manner, suggesting mRNA stabilization effects. Remarkably, clusters 11 and 12 together represent 80% of the upregulated genes analyzed in this study, which indicates a rather homogeneous behavior among the yeast genes in this positive response to heat shock. In both clusters, TR displays a strong upregulation upon stress; yet while there is a delayed but sustained increase of RA in cluster 11 (enriched in the ‘Proteasome complex’ and ‘Endoplasmic reticulum’ GO categories, see below), the RA increase in cluster 12 is faster and stronger. Although these differences may appear as minor ones, they suggest a marked divergence in mRNA stability upon heat shock between genes of clusters 11 and 12 (see below). A more stringent clustering analysis of the genes of clusters 11 and 12 resulted in four and three subgroups respectively (Fig. 3). Proteasome complex genes mostly fall grouped in the subcluster 11D in this Fig. 3, which denotes a homogeneous behavior of these GO category genes.

### Transcriptional Factor Targets Enrichment of the Clusters

There are several known TFs involved in the stress response. We looked for the enrichment in the gene targets within the clusters (#7–14) that show a positive response to stress in TR. There are some targets for Msn2/4, Hsf1 and Hac1 in clusters 7–10 that do not show a real increase in RA (not shown). Msn2/4 targets are enriched in cluster 12, specifically in subcluster 12A, whereas the gene targets for Hsf1 are enriched in cluster 11, specifically in subcluster 11A. The Hac1-resposive genes are enriched in subclusters 11C and 11D. It has been recently shown that the histone deacetylase Rpd3 participates in the activation mechanism by the Msn2/4 transcription factors [20]. Despite some of the targets of this deacetylase being present in subcluster 11A, there is a special enrichment of the targets in subclusters 12A and 12B, which coincides with the enrichment of Msn2/4. This supports the results of Ruiz-Roig et al. on the regulatory effect of Rpd3 HDAC complex on Msn2/4-dependet gene targets [20]. Thus, the RA+TR clustering is able to capture differences between the different stress-induced regulons.

### Effect of Heat Shock on mRNA Stability

We employed the absolute TR and RA values obtained by the GRO procedures to estimate the mRNA first-order degradation constants (*k_D_*, a measure of mRNA instability). For this purpose, we used a mathematical approach (see Eq. (1) in M&M) which does not assume steady-state conditions for TR and RA during the time course [3], [4]. Because *k_D_* is computed from the TR and RA absolute values, which have to be calculated by comparing external datasets, the associated error is enlarged by the mathematical manipulation. Therefore, the *k_D_* values obtained for individual genes (Table S1) are probably too noisy to allow further investigation. However, as our previous analysis showed that functionally related genes follow similar behaviors in the TR plus the RA profiles, we used average profiles of the gene groups to describe the kinetic behavior of mRNA stability. The average data for all 16 clusters are shown in Fig. S1. It should be pointed out that the represented *k_D_* values are relative to the initial (steady-state) value. Therefore, *k_D_* values over 1 denote mRNA destabilization, whereas values under 1 are indicative of stabilization effects. Clusters 2–10 mainly show destabilization for their mRNAs during most of the time course. Thus, changes in mRNA stability generally cooperate with changes in TR to increase or decrease the amount of mRNA during the heat shock response. This has been confirmed by a different analysis (see below). Clusters 7–10, however, show an antagonist effect for TR and mRNA stability.

**Figure 4:**
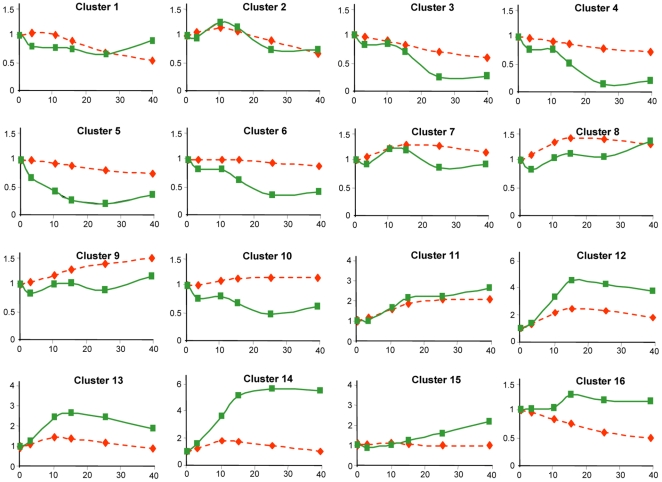
mRNA kinetics of the genes in the 16 clusters upon heat shock. RA values are represented in the *y* axis as a function of time (min) (shift from 25°C to 37°C at time 0). Experimental RA values (continuous lines) were determined as indicated in the text. Theoretical RA values (dashed lines) were determined from the experimental TR values by assuming a constant *k_D_* identical to that of time 0. Graphics represent the mean values corresponding to all the genes in the indicated cluster in relative units, referring to the mean value at time 0. Note that different scales are employed for the *y* axis depending on the cluster.

An alternative way to visualize the importance of the changes in mRNA stability in the transcriptional response to heat stress is by comparing the experimentally determined RA kinetics over time with the theoretical one (see equation [1] in M & M) obtained from TR values and the initial RA by assuming no changes in mRNA stability (Δ*k_D_* = 0). Fig. 4 represents the mean real and theoretical kinetics of the genes in each individual cluster. When comparing both kinetics profiles, it is clear that the actual kinetics from clusters 3–10 runs below the theoretical one, which is indicative of destabilization effects. Therefore, the ribosome biogenesis genes especially enriched in clusters 3 and 4 are downregulated through both the decrease in TA and the destabilization of mRNA. On the contrary, the actual RA kinetics from clusters 12–16 is above the theoretical one, indicating stabilization effects. Cluster 11, which includes the largest number of genes among the 16 clusters, as well as clusters 1 and 2, show very similar actual and theoretical kinetics, which therefore indicate modest, if any, mRNA stabilization effects modulating of the stress response in these genes. This kind of representation is, thus, particularly suited to show that some groups of genes strongly deviate from the behavior predicted from the TR changes, evidencing that either decreased mRNA stability is used to rapidly diminish mRNA levels (clusters 3–6), or that the stabilization of mRNA is used to cooperate with a TR increase to raise mRNA levels (clusters 12–14). Therefore, we can conclude that about 62% of the yeast genes undergo significant mRNA stability regulation during the heat shock response. There is a decrease in stability for downregulated genes (clusters 3–6) that cooperates with decreases in TR. Most upregulated genes (clusters 12–16) have an mRNA stabilization that cooperates with a TR increase. Thus, 36% of the genes experience homodirectional changes in TR and mRNA stability and 26% (clusters 7–10) do not. Finally, the general analysis of the correlation coefficients between the theoretical and the experimental RA data shows that generally there is a good positive correlation (see Fig. S2), meaning that TR change is the main determinant of the transcriptional response to heat stress.

### RNA Binding Protein Targets Enrichment of the Clusters

**Figure 5:**
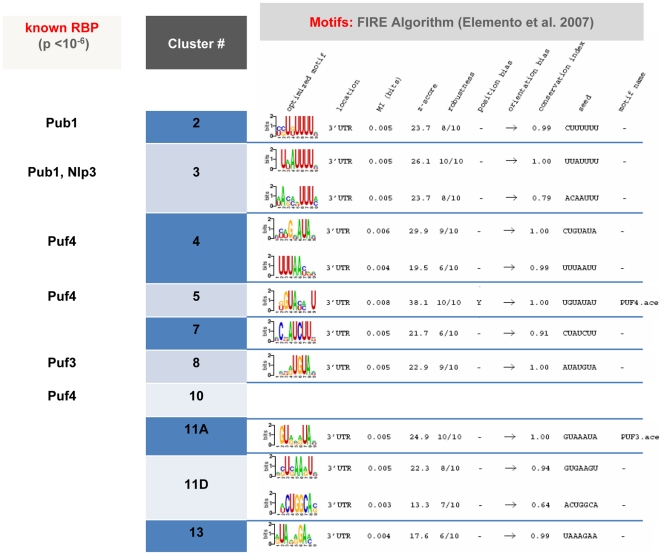
RNA binding protein (RBP) and mRNA 3′UTR motif enrichment analyses. Supervised and unsupervised analyses for RBP and mRNA 3′UTR motif enrichment, were carried out. In the left part of the panel, the *p*-values associated with the presence of the well established RBP binding motifs in the clusters are displayed. On the right, the consensus sequence motifs found in the 3′UTR of the genes belonging to the clusters are shown, as obtained by the FIRE algorithm [24]. Only those clusters with over-representation of RBPs or significant 3′UTR motifs are shown.

Since we have found groups of genes that behave co-coordinately with regard to mRNA stability, we investigated also the possible existence of RBPs, which may be responsible for this coordination. There are several known RBPs with established or predicted mRNA targets. Some of the targets have been detected experimentally by immunoprecipitation of RBP [21]–[23]. Others have been predicted by algorithms that use information from gene expression data and features of the putative motif sequence [24]. Using published data [21]–[24], we found that some of the clusters from Fig. 2 are enriched in targets or putative targets for several RBPs (Fig. 5). Using the FIRE algorithm as described by Elemento et al. [24], we also found enrichment in an unsupervised fashion for some known and unknown motifs. The most significant enrichments found are shown in Fig. 5. Pub1 targets are enriched in clusters 2 and 3. This protein has been shown to have a quite large (368 according to [23], >1000 according to [22]) number of mRNAs enriched in GOs related to translation [33], which are also enriched in cluster 3. Npl3 targets are also significantly enriched in cluster 3, coinciding with its known preference for ribosomal protein mRNAs [22]. Other significantly enriched RBP targets are those of Puf3 (cluster 8) and Puf4 (clusters 4, 5, 10). In this last case, there is again a good correspondence with the known selectivity of Puf4 for ribosome biogenesis mRNAs [22].

An unsupervised analysis using the FIRE program to look for overrepresented 3′ UTR motifs found the motifs of Puf3 (cluster 11) and Puf4 (cluster 5), as well as other new ones not assigned to known RBPs (see Fig. 5).

### Experimental Determination of mRNA Decay Rate Using the tet Promoter

The predictions on mRNA stability kinetics, as determined from the GRO analyses, were experimentally tested for some genes. For this purpose, promoters of the corresponding genes were substituted for the doxycicline-regulatable *tetO_2_* promoter. The mRNA decay rates were determined in these strains by measuring the mRNA signal levels in the Northern blots from samples taken after adding doxycycline. Previously, we showed that the repression of *tet* promoters using the activator (tTA)-repressor (tetR'-Ssn6) dual system takes place very shortly after doxycycline addition [25]. For each experiment, the mRNA decay rate was determined immediately prior to heat shock and at two different times after the shift; these times were selected based on decay kinetics according to the *k_D_* values predicted by the mathematical algorithm for each particular gene.

**Figure 6:**
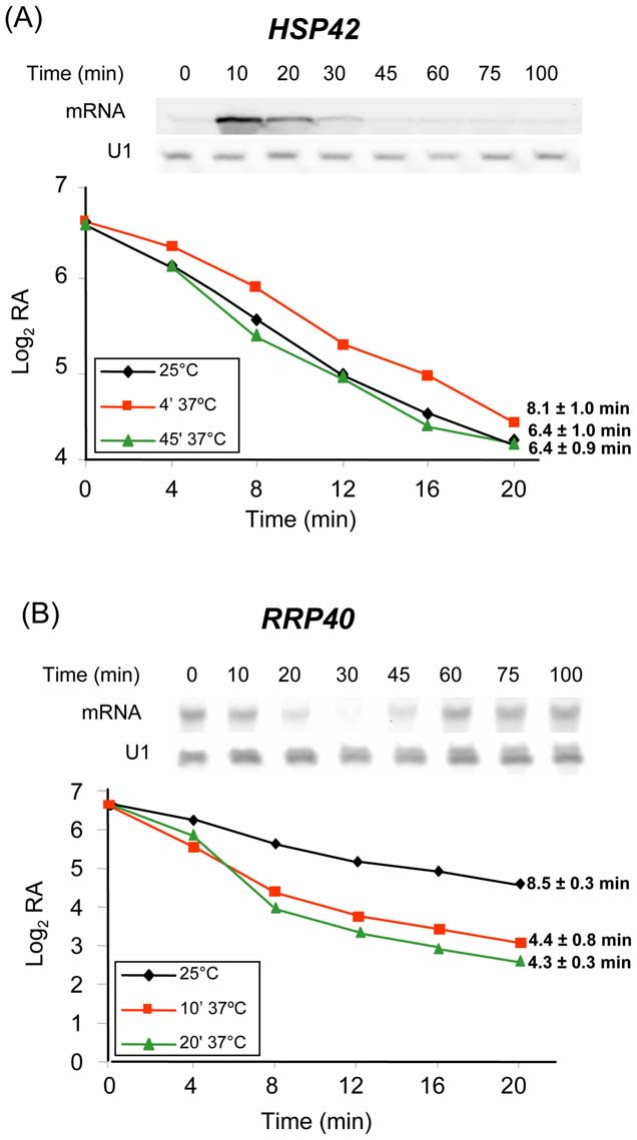
Experimental determination of mRNA half-lives before and after heat shock. Strains expressed *HSP42* (*A*, strain MML980) or *RRP40* (*B*, strain MML957) under the control of the *tetO_2_* promoter. Doxycycline (5 µg/ml) was added at time 0 to cultures growing exponentially at 25°C, or to cultures at 4 and 45 min after the shift to 37°C. In each case, aliquots were taken at time 0 and at successive times after adding doxycycline for total mRNA isolation and determination of the levels of the corresponding mRNA by Northern analysis. Graphics represent the evolution of experimentally determined relative RA on a log scale as a function of time for the representative experiment mean half-life values, and the standard deviations of a total of three independent experiments are also indicated. To determine these values, a linear regression of experimental data was calculated by considering only the initial points for which linearity was maintained. The *upper panels* show the Northern analyses of RA expressed under the respective own promoters in wild-type W303-1A cells growing at 25°C, after being shifted at time 0 to 37°C. U1 is included as the loading control. The Kruskal-Wallis statistical test was applied to show that the differences between the calculated half lives were significantly different from the others for time point 4 min (*HSP42*) and time 0 (*RRP40*) and not for the other cases.

Heat shock gene *HSP42* is found in cluster 12 (Table S2). Both TR and RA for this gene increase upon stress, as determined in the GRO experiments (Fig. 2). Nevertheless, whereas the TR levels lower to original values 15 min later, the RA values remain high steadily, until finally approaching the initial level at much later times (Fig. 2 and Table S1). This behavior is indicative of an mRNA stabilization effect (Fig. S1). By Northern analyses, we confirmed that the *HSP42* mRNA level peaked at around 10 min after the heat shock to decrease at later times (Fig. 6A, upper panel). GRO data show that increased TR precedes that of the mRNA, and it is mathematically predicted that *HSP42* mRNA molecules are transitory stabilized a few minutes after the heat shock. In this way, the transitory and modest TR induction combined with the mRNA stabilization would lead to increased mRNA levels. We employed the *tetO-HSP42* construct to confirm the above predictions. The *HSP42* mRNA half-life was determined before stress and at minutes 4 and 45 afterward. As expected, the initial mRNA decay rates followed first-order kinetics, and half-lives were calculated from the slopes of the log curves. A half-life value of 6.4 min during exponential growth increased to 8.1 min after 4 min of applying the stress, which dropped again to 6.4 min after 45 min (Fig. 6A, lower panel). Although the increase in the mRNA half-life immediately after the stress was modest, the difference was reproducible between experiments. Therefore, *HSP42* mRNA became transiently stabilized in relation to unstressed cells, thus confirming mathematical predictions. Similar results were obtained when we experimentally determined the half-lives of some other genes included in cluster 12, such as *ALD4* (data not shown).

Among the genes involved in ribosome biogenesis, we selected *RRP40* for the experimental determination of mRNA half-life before and upon heat stress. *RRP40* is in cluster 10, which shows real and theoretical RA kinetics similar to clusters 3–5 in Fig. 4. Clusters 3–5 include the majority of the ribosome biogenesis genes. Decay kinetics indicate that *RRP40* mRNA is clearly destabilized upon stress from an initial half-life of 9.5 min in non stressed cells to 4.4 and 4.3 min at post-shift minutes 10 and 20, respectively (Fig. 6B, lower panel). The results experimentally confirm that for *RRP40* (and also for *RPS6A* in cluster 4, data not shown), mRNA destabilization contributed together with TR downregulation to the decrease observed in mRNA levels upon the heat shock.

### Behavior of Relevant Gene Functional Categories upon Heat Shock

Our previous GRO studies under oxidative stress indicated a correlation between transcription parameters and gene function for several GO categories [4]. Like all stress responses heat shock induces a downregulation of translation-related gene functional groups [1], [2]. In our experiment these GO categories are concentrated in clusters 3–5. These are clusters that show a dramatic destabilization of mRNA (see Fig. 4 & Fig. S1) that contributes with TR downregulation to a rapid decrease of mRNAs. Although at first sight the parallel decrease in TR and RA curves for those genes (see Fig. S3) seems to exclude an effect of stability changes, it should be considered that kinetic laws oblige that TR changes precede the RA ones. The theoretical RA curve shows that the decrease in TR, per se, cannot explain the fast decrease in RA. In fact the k_D_ curves (Fig. S1) for those clusters show a clear destabilization. Indeed, the stability decrease of the mRNA of one of these genes, *RRP40*, has been experimentally confirmed.

**Figure 7:**
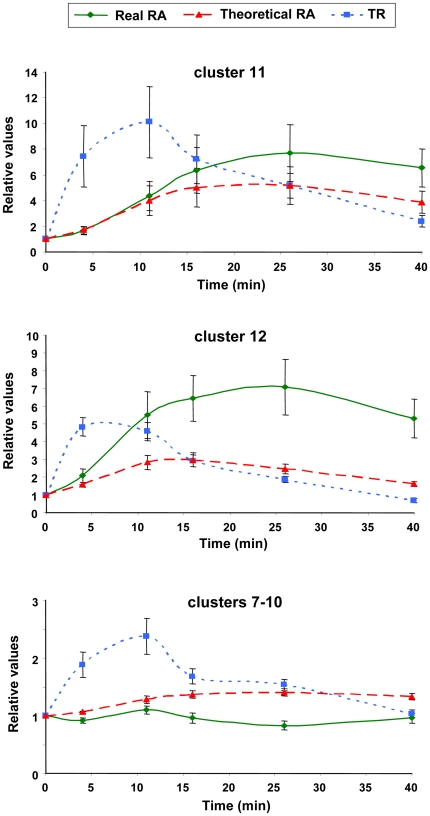
TR and real and theoretical RA values of protein folding genes after heat shock. Genes in the GO category “Protein folding” (listed in Table S3) were considered for analysis, after being subdivided between those in clusters 7–10, 11 or 12. Mean values for the three parameters were calculated and plotted as a function of time after the heat shock from 25 to 37°C. The values are represented relative to the mean value at time 0. *Bars* indicate the standard error for each time point. Theoretical RA values were calculated as indicated in the legend of Figure 4.

The other part of the stress response is the upregulation of stress-related genes. One of these gene classes involves the enhancement of the protein refolding pathway in order to reconstitute unfolded proteins. In this process some heat shock proteins play an important role [26]. We therefore analyzed our heat shock GRO data for the 67 genes of the ‘Protein folding’ GO category (Table S3). A majority of these genes are upregulated for TR and/or RA upon stress and are included in cluster 11 (21 genes) and 12 (12 genes) or dispersed between clusters 7 to 10 (18 genes). Two different patterns exist among the upregulated genes. In genes of clusters 11 and 12, both TR and RA increase upon heat shock, TR preceding RA (Fig. 7). Theoretical RA values calculated assuming a constant *k_D_* value predict a mRNA stabilization effect after the shock, which would contribute to RA increase for these protein folding genes (Fig. 7). The experimental verification of the mRNA stability change of one example of these genes, *HSP42*, has been described above. In contrast, a different behavior is observed for protein folding genes in clusters 7 to 10 (Fig. 7). Such genes display a modest increase of TR which is not paralleled by experimentally determined RA values, pointing to a sustained mRNA destabilization (Fig. 7). As expected, most of the *HSP* genes involved in protein folding display a homogeneous behavior and are in the upregulated group of genes (clusters 11 and 12) controlled by Hac1, Hsp1 and Msn2/4 TFs (Fig. 3), see Table S3.

This relative homogeneity was not observable in the GRO experiments upon oxidative stress [4]. Among the non-upregulated protein folding genes there are seven members of the *CTT* family, which code for a Ctt complex functionally related to the protein biosynthetic machinery [27], and also *PIH1*, which is involved in pre-rRNA processing [28]. This would be in accordance with the downregulation of translation-related genes observed upon heat shock, which has been previously mentioned.

In summary, our comprehensive transcriptional analysis of the heat shock transcriptional response shows that both branches of the environmental stress response, down- and up-regulated genes are contributed in a important way by changes in mRNA stability that are homodirectional with TR changes.

## Discussion

In this work we analyzed the effect of a moderate heat shock on different mRNA parameters at the whole genome level using the GRO approach. From the TR and RA experimental data, changes in mRNA stability during stress have been inferred. Thus, a global trend for mRNA stabilization is predicted upon such a stress as a general increase of RA is observed to reach levels which are then maintained at later times (Fig. 1). In contrast, Pol II-mediated TR experiences only a transient increase immediately upon stress. Therefore, mRNA stabilization effects seem to contribute to reach and maintain the increased global RA value during the time course of the experiments described herein. These data contrast with the general destabilization behavior predicted to take place as a part of the yeast response against moderate oxidative stress [4]. On the other hand, the calculation of individual gene profiles for RA and TR allows a clustering analysis using both time courses (Fig. 2). This kind of analysis permits the classification of genes according to the whole transcriptional response because it captures the changes in both mRNA synthesis (TR) and mRNA degradation. The use of TR profiles has been demonstrated to be a better tool to detect regulons [29]. In that study, the independent meta-analysis of our previously published data on carbon-source change [16], oxidative stress [4] and osmotic stress [6] responses demonstrated that nascent TR datasets predict transcriptional co-regulation better than RA profiles. In the current work, we used the RA-TR profiles and discovered that the behavior of Msn2/4-dependent genes follows a different response pattern (cluster 12A, Fig. 2 and Fig. 3) than those genes regulated by heat shock-specific factor Hsf1 (cluster 11A, Fig. 2 and Fig. 3). The average *k_D_* profile for clusters 11 and 12 (Fig. S1) also shows how initial destabilization distinguishes cluster 11 from cluster 12. This may be an unrelated but characteristic effect of Hsf1-dependent genes, or it may be a secondary effect of a transcription factor on the fate of synthesized mRNA as has been suggested for Msn2/4-dependent genes [6].

A general view of the 16 clusters shown in Fig. 2 indicates that half of the *S. cerevisiae* genes for which data have been obtained are grouped in two main clusters (11 and 12). The concentration of genes in fewer clusters than in the previous GRO study on the oxidative stress response [4], demonstrates that *S. cerevisiae* cells display a more homogeneous response for heat stress than for oxidative stress. In the oxidative stress response, an opposed effect of mRNA stability versus RA trend has been shown [17]. This apparently paradoxical behavior has been interpreted as a way to modulate the speed of the response, as previously predicted [3]. In the same study, however, the response to DNA damage stress was seen to follow homodirectional changes for RA and mRNA stability [17]. In our current results the accelerating effect of mRNA destabilization can be seen in clusters 3–5. In these clusters, genes related with translation reduce their mRNA level rapidly (Fig. S3), perhaps to prevent unnecessary expenditures in this energetically-demanding process [30]. A homodirectional response has also been found in osmotic stress [18]. In this case the authors argue that the change in mRNA stability precedes the change in RA. Using our GRO protocol in this work, we can further explore these behaviors because we obtain separate data for TR (the transcriptional response itself) and RA (the consequence of the transcriptional response modulated by changes in mRNA stability). We see here that this stress induces mainly homodirectional changes because all the upregulated genes show mRNA stabilization profiles (clusters 11–16), and most of the downregulated genes (clusters 2–6 and 10) present mRNA destabilization. Thus, we confirm that most of the homodirectional changes (as suspected) between RA and mRNA stability are due to the homodirectional behaviors of TR and mRNA stability. However, in about one quarter of the genes, clusters 7–10, mRNA degradation counteracts the TR trend, provoking even an opposite profile between TR and RA (cluster 10). In relation to the anticipation of the mRNA stability response seen by Molin et al [18] in osmotic stress, our current data show that the destabilization peak in most clusters of the upper branch takes place between 15 and 20 min (Fig. S1), whereas the minimum in RA appears later (Fig. 2). In the genes of the lower part of the tree (Fig. 2), mRNA stabilization peaks at min 5–10, clearly before the RA (which peaks at the end of the time course, or even later). This agrees with the proposal of Molin et al [18]. However, it should be considered that an advance of the TR peak over the RA one may naturally result from kinetic laws and does not necessarily mean an advanced cell response.

Among the genes which accumulate mRNA as a result of heat shock (clusters 11–16), different TR kinetics are observed. Thus, the small number of genes included in clusters 15 and 16 displays stable or decreased TR, indicating that RA upregulation results exclusively from mRNA stabilization effects. This situation would have remained unnoticed in standard DNA microarray analyses. Remarkable stabilization is also predicted for the genes of cluster 14, for which RA upregulation (about 4- or 5-fold) is much higher than that predicted from its TR profile (Fig. 4). Other previously undetected behaviors correspond to those genes with a stable RA profile whose transcription, however, has clearly altered, as in clusters 1–2 and 7–9. They represent apparent futile behaviors in which changes in mRNA stability are devoted to compensate changes in TR (as mentioned above). This behavior was also observed in our previous work on the oxidative stress response [4]. To date, we can provide no explanation for it, but it is interesting to point that another kind of apparent futile pathway has been observed by Warringer et al [31], who found that some transcriptionally induced mRNAs are not translated at all during the osmotic stress response. These results suggest the existence of still unknown gene regulatory strategies which are not just the straightforward ones [32]. In any case, and as previously suggested [3], the increases in both the transcription and degradation rates for mRNA can be a way to speed the response. On the other hand, the simultaneous decrease in both rates can be used to save energy dispenses. Therefore, we conclude that the heat shock response seems to be contributed both by mRNA transcription and stabilization effects. This is particularly relevant in the case of genes involved in protein refolding (Fig. 7).

It has been proposed that the existence of genes commonly regulated at the mRNA stability level (post-transcriptional regulons, [33]] is due to the existence of RBPs that affect the stability of their mRNA targets [21], [22]. Those proteins have been experimentally studied by performing immunoprecipitation and microarray analysis of the RNA bound fraction [21]–[23]. Their RNA sequence binding motifs have been determined either experimentally *in vitro* or *in vivo* [21]–[23] or by computer searches using newly developed algorithms [21]–[24]. Here we have performed, for the first time, a search for overrepresented RBPs motifs in clusters of genes that are characterized by having a common mRNA stability profile during a time course. We have found that some of the gene clusters include statistically overrepresented Pub1, Npl3, Puf3 and Puf4 targets (Fig. 5). This is an additional proof of the existence of post-transcriptional regulons with an overlapping effect on gene regulation with classical transcription regulons. Our analysis has found some putative new binding motifs in other possible post-transcriptional regulons and thereby opens new paths for further research.

However, not all the genes are modulated at an important level by mRNA stability. Clusters 2 and 11 (with 35% of the analyzed genes) show almost identical experimental and theoretical RA profiles (Fig. 4). The number of genes with a high positive correlation between the theoretical RA (assuming no stability changes, see M & M) and the experimental RA (Fig. S2) is large, supporting that TR changes are the main cause of RA changes upon heat stress.

Finally, we wish to point out that the discrepancies between the profiles of RNA polymerase activity (TR obtained by run-on labeling) in genes, and the RA during the stress response, mainly reflect the post-transcriptional effect of mRNA stability. Our current and previous results [4], [6], [15], as well as the results from many other laboratories [5], [17], [18], [33], demonstrate that changes in mRNA stability are a strong component of the stress response. However, a recent publication [34] indicate that the discrepancies between changes in the mRNA and Pol II presence (based on immunoprecipitation experiments) in genes are due to non productive (cryptic) transcription. Kim et al. [34] argue that the genes for which an excess of mRNA is produced, regarding the presence of the Pol II molecule, were often associated with the existence of overlapping non coding mid-log-expressed transcripts. Although this is statistically shown in their analysis, this work does not demonstrate the existence of a cause-effect relationship. Their argument also involves additional speculation that non productive transcription is repressed during the stress response, which has not been demonstrated. In fact, we have found that the presence of cryptic transcription has little effect on a gene in the discrepancy between the experimental and theoretical RA profiles (JG-M and JEP-O in preparation). Although the effect can be more important for particular genes with a high proportion of antisense transcripts, it cannot account for most of the discrepancies detected between mRNA and Pol II changes. Therefore, it seems more straightforward and more experimentally supported to consider the discrepancies between the TR and RA profiles to the result of the delay required by kinetic laws, and also of the changes in mRNA stability.

## Materials and Methods

### Strains, Plasmids and Growth Conditions

*S. cerevisiae* wild-type strain W303-1A (*MAT* **a** *ura3-1 ade2-1 leu2-3,112 trp1-1 his3-11,15*) and its derivative MML830 were used. The MML830 strain [4] was obtained by the chromosomal integration of linearized plasmid pCM244, which codes for the doxycycline-inducible *tetO_2_* promoter repressor tetR'-Ssn6. After employing the promoter substitution cassette from plasmid pCM224 [35], the following strains were obtained in which the endogenous promoters of several genes in MML830 were replaced with the *tetO_2_* promoter: MML957 (*tetO_2_-RRP40*), MML980 (*tetO_2_-HSP42*), and MML1042 (*tetO_2_-ALD4*). MML896 is a MML830 derivative with the *rps6A::kanMX4* and *rps6B::natMX4* deletions and is transformed with plasmid pMM803. This is a *URA3* centromeric plasmid which derives from pCM188 [36] and contains the *RPS6A* open reading frame, plus 339 bp of the 3′-UTR region of *RPS6A*, under the control of the *tetO_2_* promoter.

Cells were grown in rich YPD medium (1% yeast extract, 2% peptone, 2% glucose) or in defined SC-glucose medium [37]. Doxycycline at 5 µg/ml was added to repress genes under the *tetO_2_* promoter. Cultures exponentially growing in liquid medium (1.5–2×10^7^ cells/ml) at 25°C were heat-shocked by adding an equal volume of fresh medium at 49°C, to immediately reach a final temperature of 37°C, and by keeping them in a bath at 37°C during the sampling interval. Samples were taken immediately before changing the temperature (time 0) and at 4, 11, 16, 26 and 40 minutes afterward.

### Genomic Run-on

GRO analyses (three independent experiments) were done as in Ref. 16, with the modifications described in Ref. 4. With this procedure, we obtained the RA and TR measurements for each gene and each time point. Total RNA and poly(A) mRNA per cell were measured as in Ref. 16. Total TR for Pol II was determined by summing all the individual spots present in the macroarray for the genes transcribed by RNA polymerase II [16]. The individual TR values obtained from the GRO analyses are based on the determination of the number of RNA polymerase complexes transcribing each open reading frame at the time of sampling. However, the changes in the nucleotide incorporation rates, which may occur *in vivo* when the stress conditions are applied, are not usually considered. In the particular case of heat shock, the temperature shift from 25°C to 37°C is expected to increase the nucleotide polymerization rate substantially. In the absence of direct experimental data about temperature dependence of *S. cerevisiae* RNA polymerase, we considered available values of 13 Kcal/mol [38] and 9.7 Kcal/mol [39] for the apparent activation energy of the elongation step in the case of the *Escherichia coli* RNA polymerase. By assuming Arrhenius dependence of the catalytic constant, these values predict the elongation speed to roughly double as a result of the temperature shift. Therefore, the GRO-determined TR values (except those of time 0) were multiplied by two for further calculations.

### Messenger RNA Decay Rate Calculations

RAs were assumed to be in the steady state at the onset of stress. Therefore, the initial (steady-state) decay rate (*k_D_*) was calculated as the ratio of the TR to RA values determined at time 0 [16]. After the onset of stress, under (presumably) non steady-state conditions, the decay rates were inferred from the experimental values of TR and RA provided by the GRO technique throughout the time course. By assuming a linear variation of TR in between the experimentally determined values, the following relationship among TR, RA, and *k_D_* has been postulated [3]:
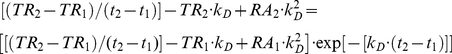
(1)where TR_1_ and TR_2_, and RA_1_ and RA_2_, are the experimentally determined values for TR and RA at the successive time points t_1_ and t_2_. By numerically solving for *k_D_* (using a bisection algorithm written as a Visual Basic for Applications program in a Microsoft Excel spreadsheet) in the above equation, a mean *k_D_* value for the time interval between t_1_ and t_2_ was obtained and assigned to a time point in the middle of the interval between the two experimental times. In contrast, to highlight the effect of stability modulation, the same equation has been used to determine the expected RA values at different times from an initial RA amount and the experimental TR values by assuming a constant k_D_ equal to the steady-state value observed at time 0.

### Clustering Procedures

The changes in TR and RA for all the yeast genes were evaluated by a cluster analysis of the normalized averaged values. For the cluster analysis of the results, we used the SOTA Tree server included in the Gene Expression Pattern Analysis Suite v 3.1 (http://www.gepas.org) located on the web server of the CIPF Bioinformatics Unit (cluster conditions as in [4]). To test the potential enrichment in the GO categories in the different groups obtained in this study, we used the FuncAssociate (http://llama.med.harvard.edu/cgi/func/funcassociate), which uses a Monte Carlo simulation approach and accepts only significant GO categories according to the adjusted *p* value computed from the fraction of 1000 simulations under the null-hypothesis with the same or a smaller *p* value and after correcting for multiple simultaneous tests.

### Transcription Factor and RNA binding Protein Analyses

Targets for the TF Msn2/4, Hsf1 and Hac1 and the histone deacetylase Rpd3 were taken from [26], [40], [41]. Significance of the enrichment of these targets in the clusters obtained by the SOTA server (see previous section) was evaluated by means of a binomial distribution test. Only the *p*-values lower than 10^−6^ are shown. RNA binding protein analyses were conducted in two ways. First, a supervised analysis was done by testing the enrichment of the different clusters in the targets of several well established RBP, such as Pub1p, Nlp3p, Puf3p, or Puf4p [21], [22], using a binomial test. Second, we did an unsupervised analysis by using the FIRE server [24] which helps finding overrepresented sequence motifs in the 3′UTR regions of the genes belonging to a given cluster. Only those clusters with overrepresented 3′UTR sequence motifs were considered.

### Northern Blot Analyses

RNA electrophoresis, probe labeling with digoxigenin, hybridization and signal detection in a Lumi-Imager equipment (Roche Applied Science) were done as previously described [25]. Gene probes were PCR-generated from genomic DNA using oligonucleotides designed to amplify internal open reading frame regions. Background values were determined for a region lacking a visible signal of the same size as the measured band and adjacent to it. This background was subtracted from the respective band signal value.

### Accession Numbers

All the experiments were done in triplicate. Genomic data are stored in the Valencia Yeast (VYdBase; http://vydbase.uv.es/) and the GEO databases. The GEO accession number for the whole experiment TR and RA dataset is GSE24488.

## Supporting Information

Figure S1 **Predicted** ***k_D_*** **kinetics for the different gene clusters upon heat shock.** *k_D_* values are represented in the y axis as a function of time (min) (shift from 25°C to 37°C at time 0). Graphics represent the mean *k_D_* value corresponding to all the genes in the indicated cluster in relative units referring to the mean *k_D_* value at time 0. The horizontal line marks the time 0 unit level. Bars represent the standard error for each time point. Two different *k_D_* scales are employed, depending on the cluster. Note that because the y scale is natural (not logarithmic), the *k_D_* increases (ratios >1 as regards time 0) are apparently expanded in relation to the *k_D_* decreases (ratios <1). Note also that the time points for the calculated *k_D_* correspond to the mid point between two experimental time points (see Materials and Methods).
(PDF)

Figure S2 **R (Pearson) correlation coefficient between theoretical and experimental RA.** Histograms of the Pearson coefficient (R) for the correlations of individual gene values of the predicted theoretical mRNA amount (RA) using equation 1 and a constant *k_D_* (equal to the one at time 0) versus the experimental RA data. The mode r value for the yeast genes is marked with an arrow.
(PDF)

Figure S3 **TR and real and theoretical RA values for ribosomal protein genes and rRNA processing genes after heat shock.** Genes in both GO categories (listed in Table S3) were considered for analysis, Mean values for the three parameters were calculated and plotted as a function of time after the shift from 25 to 37°C. The values are represented relative to the mean value at time 0. *Bars* indicate the standard error for each time point. Theoretical RA values were calculated as indicated in legend of Figure 4.
(PDF)

Table S1 **Parameter (RA, TR and k_D_) absolute values for all the genes.**
(XLS)

Table S2 **List of genes included in each cluster from** **Figure 2** **.**
(DOC)

Table S3 **Parameters (real RA, theoretical RA and TR) for the genes of the GO categories ‘Protein Folding’, ‘Ribosomal Protein’ and ‘rRNA Processing.’**
(XLS)
