# Supplementary figures and images for: Heat Shock Response in Yeast Involves Changes in Both Transcription Rates and mRNA Stabilities
Source: PLoS One. 2011 Feb 25;6(2):e17272. doi: 10.1371/journal.pone.0017272 (PMC3045430; doi:10.1371/journal.pone.0017272)

Figure S1

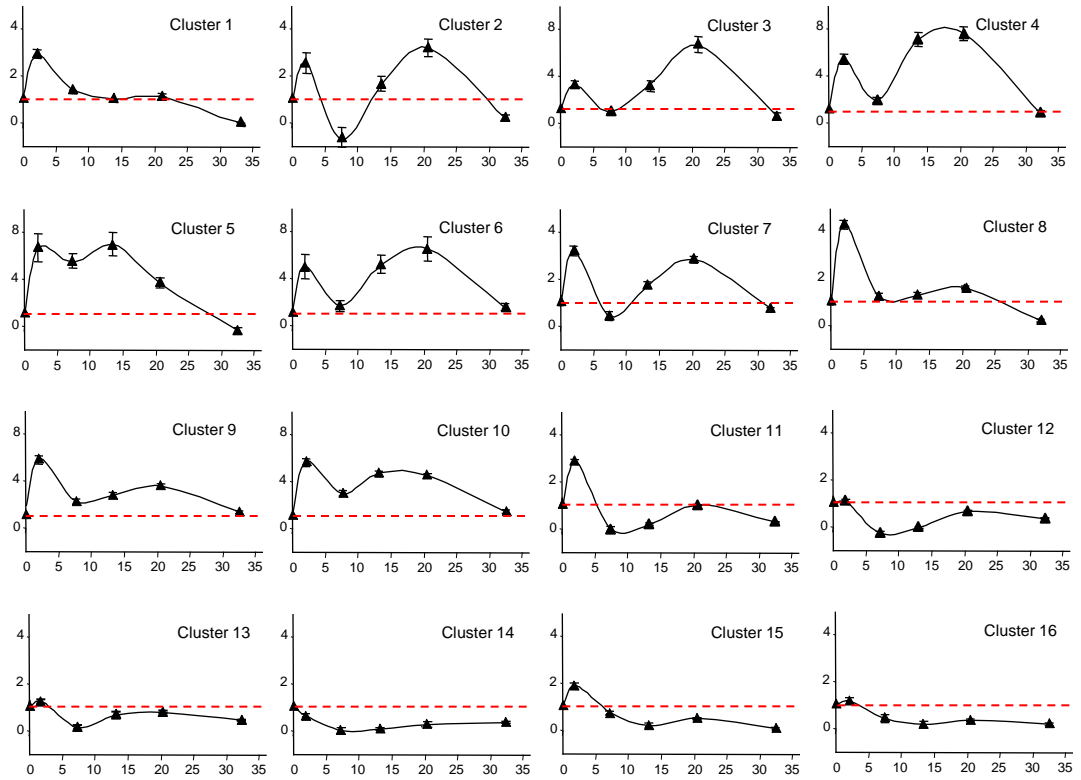

Supplement: Figure S1 — Predicted kD kinetics for the different gene clusters upon heat shock. kD values are represented in the y axis as a function of time (min) (shift from 25°C to 37°C at time 0). Graphics represent the mean kD value corresponding to all the genes in the indicated cluster in relative units referring to the mean kD value at time 0. The horizontal line marks the time 0 unit level. Bars represent the standard error for each time point. Two different kD scales are employed, depending on the cluster. Note that because the y scale is natural (not logarithmic), the kD increases (ratios >1 as regards time 0) are apparently expanded in relation to the kD decreases (ratios <1). Note also that the time points for the calculated kD correspond to the mid point between two experimental time points (see Materials and Methods). (PDF) [file pone.0017272.s001.pdf]

Figure S2

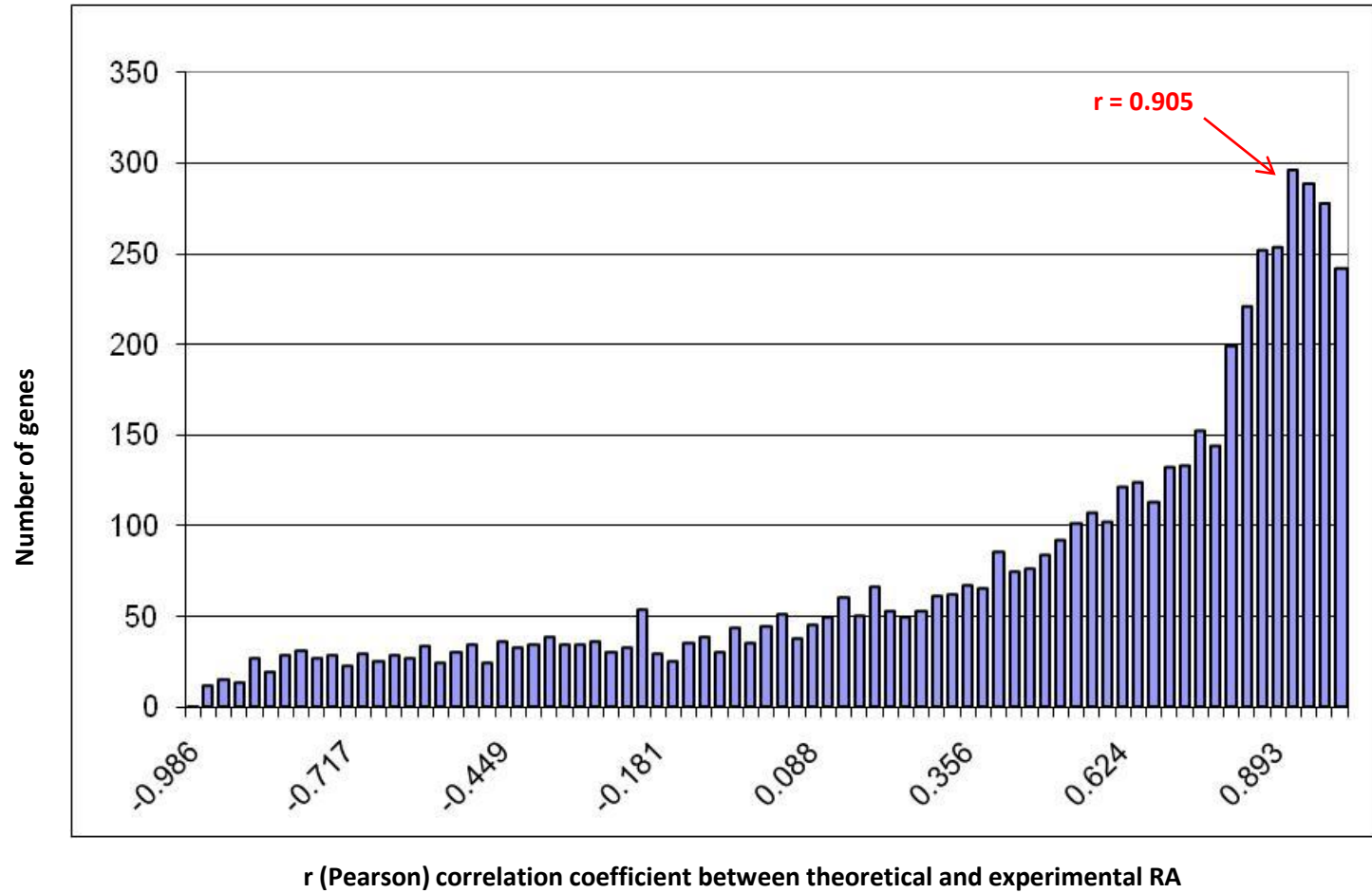

Supplement: Figure S2 — R (Pearson) correlation coefficient between theoretical and experimental RA. Histograms of the Pearson coefficient (R) for the correlations of individual gene values of the predicted theoretical mRNA amount (RA) using equation 1 and a constant kD (equal to the one at time 0) versus the experimental RA data. The mode r value for the yeast genes is marked with an arrow. (PDF) [file pone.0017272.s002.pdf]

Figure S3

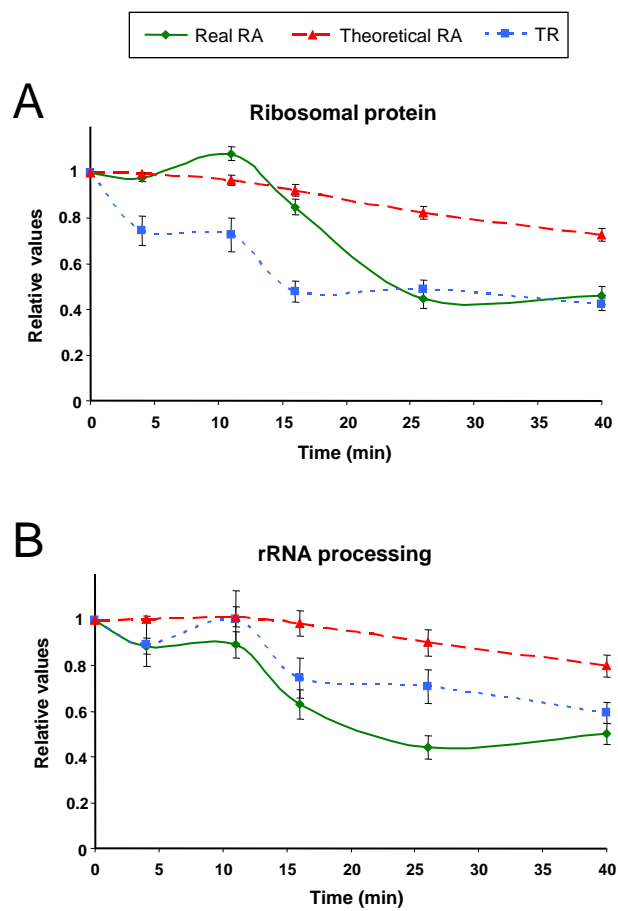

Supplement: Figure S3 — TR and real and theoretical RA values for ribosomal protein genes and rRNA processing genes after heat shock. Genes in both GO categories (listed in Table S3) were considered for analysis, Mean values for the three parameters were calculated and plotted as a function of time after the shift from 25 to 37°C. The values are represented relative to the mean value at time 0. Bars indicate the standard error for each time point. Theoretical RA values were calculated as indicated in legend of Figure 4. (PDF) [file pone.0017272.s003.pdf]
