# Supplementary material for: Heat Shock Response in Yeast Involves Changes in Both Transcription Rates and mRNA Stabilities
Source: PLoS One. 2011 Feb 25;6(2):e17272. doi: 10.1371/journal.pone.0017272 (PMC3045430; doi:10.1371/journal.pone.0017272)
Supplement: Table S2 — List of genes included in each cluster from Figure 2 . (DOC) [file pone.0017272.s005.doc]

Cluster1(node18)

Thereare179elements.

ACO1

ALK1

ALR1

APP2

ARP6

ARP7

ASE1

ATS1

BOI2

BRE4

BRF1

BSP1

BUD32

BUD4

BUD6

BUD8

CDC123

CDC14

CDC43

CDC5

CIN1

CKB1

CKB2

CLA4

CLB1

CNA1

CWC27

DAN4

DBF20

DCI1

DHR2

DPB4

ERC1

ESS1

FAA4

FCY2

FHL1

FUI1

GIC1

GLN1

GUP2

HCM1

HEM4

HFI1

HMG2

HNM1

HOT1

HTB1

HXT4

IES2

ISC10

ISU2

ITR1

KCC4

LCB3

LEM3

LEU2

LEU9

MAK31

MCM2

MDM1

MED1

MET7

MIA1

MID2

MKK1

MNL1

MOB1

MRE11

MRP13

MRPL11

MRPS5

MRS1

MRS6

MTM1

NDE1

NGL2

NMD2

NOT3

NPL6

NUP1

NUP2

NUP57

NUP84

OAC1

PDS5

PEX7

PHO81

PHO91

PRP24

PRP31

RHB1

RSM25

RSM7

SAM3

SAN1

SAP30

SCS7

SET1

SFH1

SFL1

SGV1

SKN1

SKN7

SMM1

SNL1

SRB2

SRO7

SRP72

STE20

STE4

STE50

STH1

SWI5

THP2

TIM13

TOS4

TPO3

TRF4

TRI1

TRS20

TRS23

TSC10

UIP5

VAM3

VAN1

VIP1

VPS75

WSC3

YAE1

YAP7

YBL053W

YCL005W

YCL022C

YCR015C

YDR049W

YDR381C-A

YDR444W

YEL077C

YFL067W

YFR055W

YGL114W

YGL139W

YHL029C

YHL050C

YHP1

YHR121W

YIL092W

YIR035C

YJR018W

YJR146W

YKL018C-A

YKL027W

YKR077W

YLR125W

YLR358C

YLR440C

YML023C

YML025C

YML053C

YML099W-A

YMR147W

YMR171C

YMR193C-A

YNG1

YNL050C

YOL138C

YOR073W

YOR112W

YOR166C

YOR205C

YOR366W

YPL017C

YPL073C

YPL112C

YPR004C

YPR114W

YPR123C

ZRT2

Cluster2(node17)

Thereare156elements.

ACS2

ADH3

AGP1

ALD5

ASK1

AST1

ATP16

AYT1

BAP2

BAP3

BUD14

CBF1

CCR4

CCW14

CDC47

CHS2

CLN3

COX19

DIP5

DUT1

ECM14

ELF1

ELO1

ERG1

ERG2

FET4

FKH2

FLR1

FUS3

GAP1

GAS5

GCN20

GCN4

GIM3

GLT1

GLY1

GND1

GNP1

GPX2

GZF3

HAP4

HDA1

HEM12

HHT1

HIS6

HMLALPHA1

HOM2

HTA1

HTA2

HTB2

ILS1

ILV2

IMD1

IPP1

KAP104

KRE6

LYS2

MAP2

MCH5

MLC1

MPS1

MTR10

MUC1

NAF1

NAP1

NCB2

NSL1

NUP82

OLE1

PAN6

PAT1

PBP1

PCL7

PCL9

PET494

PET9

PHO89

PIN4

PPM1

PRS2

PTR2

PXR1

RET1

RHO3

RIB4

RIF1

RIM2

RPA14

RPI1

RPL31B

RRP7

RSC6

RTT107

SCS2

SHE1

SHR5

SIR2

SMI1

SOK2

SPE2

SPR6

SRP40

SRP54

SSB1

SSY5

SUR2

SYG1

TAT1

TEC1

TEM1

TFP3

TIM17

TIP20

TOP2

TOS1

TSM1

TUB1

URA3

UTP21

VAC17

VMA2

VMA4

WSC2

WWM1

YBL100C

YBR028C

YBR075W

YBR281C

YCK2

YDL187C

YDL241W

YDR119W

YDR133C

YDR134C

YDR352W

YDR491C

YDR492W

YDR527W

YER137C

YGR068C

YGR251W

YGR259C

YJL123C

YJR014W

YKL077W

YLR179C

YLR198C

YMR075C-A

YOL137W

YOR169C

YOR218C

YOR246C

YPL068C

YPL199C

YPR063C

ZRC1

Cluster3(node12)

Thereare262elements.

ADE6

AFG2

AMN1

APT2

ARD1

ASC1

ASH1

ATO3

BAR1

BAS1

BFR2

BMS1

BUD19

BUD22

CGR1

CLB2

CLN2

CTR1

CTS1

CUP5

CWP2

CYS4

DBP2

DBP5

DCD1

DRS1

DSE1

DSE2

DSE4

DUS4

ECM16

EGD1

EGD2

EGT2

ELP3

ENP2

ERG11

ERG3

ERG5

ERG6

EXG1

FAA3

FLX1

FPR3

FPR4

FRE7

FRS1

FUR1

FYV5

GCD2

GDS1

GIN4

GLN4

GPA1

HXT3

IKI3

IMD2

IPI2

KAP123

KEL3

KIN4

KRE33

LAS1

LSM3

LTV1

MAE1

MAP1

MIG2

MIS1

MPP10

MRD1

MSG5

NEW1

NMD3

NOC2

NOG2

NOP4

NRD1

OAR1

PAC10

PMA1

POL5

PRP11

PRS4

PUS4

RCL1

RIA1

RKI1

RNR1

RPA135

RPB8

RPC53

RPC82

RPG1

RPL10

RPL11B

RPL12A

RPL13A

RPL13B

RPL14A

RPL14B

RPL15A

RPL15B

RPL16A

RPL16B

RPL17A

RPL19A

RPL19B

RPL20B

RPL21A

RPL21B

RPL22A

RPL23A

RPL23B

RPL24A

RPL25

RPL26A

RPL26B

RPL27A

RPL27B

RPL28

RPL2A

RPL31A

RPL32

RPL33A

RPL33B

RPL34A

RPL34B

RPL35A

RPL35B

RPL36A

RPL37A

RPL37B

RPL40A

RPL40B

RPL42A

RPL43A

RPL5

RPL6A

RPL6B

RPL7A

RPL7B

RPL8A

RPL8B

RPP0

RPP1

RPP1A

RPP1B

RPP2A

RPP2B

RPS0B

RPS10A

RPS10B

RPS11A

RPS11B

RPS12

RPS13

RPS14A

RPS16B

RPS17B

RPS18A

RPS18B

RPS1B

RPS20

RPS21A

RPS21B

RPS22A

RPS22B

RPS26A

RPS26B

RPS27B

RPS28B

RPS29B

RPS3

RPS30B

RPS31

RPS31B

RPS4A

RPS5

RPS7A

RPS7B

RPS9A

RPS9B

SAM1

SAP185

SCW10

SCW11

SDA1

SEN1

SES1

SHU1

SIM1

SKI6

SMY2

SPO12

SRO9

STE2

STE6

STM1

SUP35

SWE1

SXM1

TEA1

TIF11

TOM20

TRM3

TSR1

TSR2

URA1

UTP18

UTP22

UTR2

VIK1

VPS69

YAR064W

YBL004W

YBL028C

YBL062W

YBL070C

YBL071C

YBR187W

YBR238C

YBR242W

YBR271W

YCL046W

YDL063C

YER007C-A

YGL102C

YGR021W

YGR025W

YGR035C

YGR130C

YHB1

YHR020W

YHR149C

YHR203C

YIL019W

YJL200C

YJL207C

YJL215C

YJR124C

YKL014C

YKR075C

YLL034C

YLL044W

YLR076C

YLR339C

YLR407W

YLR413W

YMR132C

YNR025C

YNR054C

YOL093W

YOR271C

YOR277C

YOR315W

YOR342C

YPL030W

YPL142C

YPL183C

YPL207W

YPR044C

Cluster4(node14)

Thereare181elements.

ADK1

APT1

ARO4

BUD20

BUD21

BUD27

CAF130

CAF20

CBF5

CSL4

DBP10

DBP3

DBP8

DBP9

DED81

DIP2

DYS1

EFT1

EFT2

ENP1

ERB1

FAR1

FAS1

FKS1

FRS2

FUN11

FUN12

GCD11

GCN1

GIS2

GUA1

HAS1

HCA4

HIR3

ILV3

IMD3

IMP3

IMP4

IPI3

KRI1

KRR1

KTI11

LCP5

LEU3

LSG1

MAK5

MES1

MFA2

MSB2

MTR4

NAN1

NIP1

NOC3

NOG1

NOP1

NOP14

NOP16

NOP2

NOP58

NOP6

NOP7

NRP1

NSA1

NSA2

NUC1

NUG1

PHM6

PHO11

PHO12

PHO5

PPA1

PPM2

PPT1

PRM6

PRT1

PSE1

PUS1

PWP1

RAD3

RHK1

RLI1

RLP7

ROK1

RP18B

RPA43

RPC34

RPC40

RPF1

RPL11A

RPL12B

RPL18A

RPL1A

RPL1B

RPL20A

RPL24B

RPL2B

RPL3

RPL30

RPL42B

RPL4A

RPL4B

RPL9A

RPL9B

RPS0A

RPS15

RPS17A

RPS19B

RPS1A

RPS2

RPS23B

RPS24A

RPS24B

RPS27A

RPS28A

RPS6A

RPS6B

RRB1

RRP1

RRP12

RRP3

RRP6

RRS1

RSA3

RVB1

SAM4

SAS10

SHQ1

SIK1

SNU13

SSB2

SSF1

SSZ1

STT4

SVS1

TEF4

TIF1

TIF34

TIF6

TPI1

TRF5

URA5

URA7

UTP13

UTP14

UTP19

UTP5

UTP7

UTP8

UTP9

VAS1

YBL077W

YBL083C

YCR016W

YCR072C

YDR324C

YDR341C

YDR417C

YEF3

YER049W

YGL245W

YGR081C

YIL091C

YJL184W

YJR041C

YKL099C

YLR022C

YLR051C

YLR419W

YML047W-A

YMR093W

YNL114C

YNL174W

YNL247W

YNR071C

YNR073C

YOL092W

YOL154W

YOR004W

YPL044C

YTM1

ZUO1

Cluster5(node15)

Thereare116elements.

ALA1

BIO2

BRX1

BUD23

BUD9

CDC20

CIC1

DAT1

DPH2

DUS3

EBP2

EMG1

FIR1

FOL1

FUN30

FYV15

GCD7

GEA1

GFD2

GLR1

HGH1

HMS2

HMT1

ILV5

LOC1

MAK11

MAK3

MAS6

MED6

MET31

MGM101

MNT2

MRPL37

MTO1

MTR3

NHP2

NNF1

NOP15

OGG1

ORC6

PHO84

PHO90

POL2

PRS3

PUS6

PUS7

RFC4

RPA12

RPB3

RPC19

RPC37

RPF2

RPL24

RPO31

RRP9

SCH9

SCM4

SEC14

SMD1

SPB1

SPE4

SRP102

SST2

STE12

SUA5

SYF2

TAF14

TFB2

TGS1

TIF3

TIF5

TRM7

UAF30

YAR070C

YBR262C

YBR266C

YBR267W

YCR043C

YCR060W

YDR339C

YDR412W

YDR413C

YGL235W

YGR160W

YGR272C

YHL013C

YIH1

YIL127C

YJL009W

YJR003C

YJR070C

YJR071W

YJR072C

YLR003C

YLR073C

YLR243W

YLR363W-A

YML009W-B

YML018C

YMR144W

YNL087W

YNL227C

YNL228W

YNR024W

YOL022C

YOL101C

YOR021C

YOR145C

YOR146W

YOR252W

YOR309C

YOX1

YPL108W

YPL146C

YPR142C

YPR143W

Cluster6(node16)

Thereare116elements.

AAH1

ACC1

ANP1

ARO1

ARO7

ARO8

CCA1

CDC33

CHO2

CPT1

CTR9

CWH36

DBP7

EMP70

ERG25

GCN3

GDA1

GIR1

GPI15

GRS1

GRX4

HIP1

HIS7

HTS1

ILV6

KIN3

KRS1

LAC1

LYS12

LYS20

LYS4

MET2

MET6

MLC2

MSB1

MSH1

MYO5

NAT1

NCL1

NCP1

NUP133

NUP145

NUP85

PFD1

POL12

POP5

PRS1

PRS5

PWP2

RAI1

RIB3

RNA1

RPA34

RPB10

RPB5

RPC11

RPE1

RPO26

RPS23A

RPS25A

RPS8A

RRP8

RSC8

SAH1

SAM2

SCP160

SHM1

SLN1

SPS2

SRL1

STT3

SUI2

SUR7

TAF9

TAH18

TCI1

TEN1

TIF2

TKL1

TOM40

TRL1

TRM1

VMA8

VPH1

VTC2

VTC4

YBL081W

YBR030W

YBR074W

YBR094W

YCR087W

YCR090C

YDL152W

YDR020C

YDR101C

YDR112W

YDR198C

YER187W

YGL232W

YGR050C

YHL039W

YHM2

YHR036W

YIL064W

YIL158W

YJL202C

YLR016C

YLR145W

YMR290W-A

YMR310C

YNL300W

YNR018W

YNR046W

YOR091W

YOR248W

ZRT1

Cluster7(node20)

Thereare330elements.

ACH1

ALO1

APD1

APE3

APL3

APL6

APM3

APQ13

ARC40

ARE1

ARG80

ARG81

ARR2

ASN1

ATF2

ATM1

ATP1

ATP14

ATP18

ATP19

ATP2

ATP3

BNI5

BRE1

BUB1

BUD28

CAF120

CAJ1

CDC1

CDC42

CHA1

CIN2

CLB5

CLN1

COR1

COX7

CUS1

CWC2

CWH43

CYS3

DAD2

DAL3

DAL81

DID2

DID4

DIS3

DJP1

DLD3

DNF2

DOT6

DPB11

DPS1

DRE2

EBS1

ECM17

ECM33

EFB1

EHT1

ERG9

ERP3

ERP4

FAL1

FCY1

FOL2

FPR1

FRE2

FRE4

FTR1

FUM1

GAS3

GCS1

GIC2

GIR2

GSH1

HDR1

HHF1

HHF2

HHO1

HMO1

HNT2

HOM3

HSL7

HXT2

HYP2

IDI1

JIP5

KAE1

KAP120

KCS1

KGD2

KRE2

KRE21

LAT1

LCB2

LEU4

LRP1

LTE1

LYS14

MDM39

MDN1

MDV1

MGA2

MLF3

MMM1

MMT1

MNN11

MRP1

MRPL24

MRPL31

MRPL35

MRPL49

MSN4

MUM2

MVD1

MXR1

NAM7

NET1

NNT1

NPT1

NUP159

NUP192

NUP42

ODC1

ORC2

OSH7

PDC2

PDR12

PDR16

PDR17

PDR5

PEP3

PEX11

PFK27

PHO8

PHO85

PKC1

PLP2

PMP1

PMT4

PNP1

POL1

POM34

POP2

POR2

PRM7

PRO3

PRP19

PRP6

PRR1

PSP1

PSP2

PSR1

PTC4

PTH2

RAP1

RCY1

RDH54

RDI1

RFC1

RHO1

RMD1

RNQ1

RPB7

RPB9

RPR42

RPS8B

RRN6

RRP14

RSM10

RSP5

RSR1

RTF1

RVB2

SAG1

SAS3

SBE22

SBH2

SEC31

SEC4

SEN34

SGM1

SGN1

SHO1

SHS1

SIP1

SIT4

SKI8

SKS1

SLS1

SNF4

SPA2

SPE1

SPL2

SPO73

SPP41

SPT15

SPT5

SRL2

SRM1

SRP101

SRT1

SSO2

STB1

SUT2

SVL3

SWI3

SYF1

TAD1

TAF12

TFG1

TFP1

THI80

TIF35

TIF4632

TOK1

TPD3

TPK3

TPO2

TRP3

TRR1

TRX1

TUB4

TUP1

TYS1

UBA2

UBC12

UBC9

UBP13

VCX1

VHT1

VMA13

VMA21

VMA7

VPS1

VPS53

VTA1

VTC1

YAR009C

YBL036C

YBR025C

YBR159W

YBR174C

YBR178W

YDL062W

YDL176W

YDR051C

YDR063W

YDR111C

YDR267C

YDR275W

YDR326C

YDR396W

YDR437W

YDR509W

YDR526C

YER076W-A

YER113C

YFR006W

YFR018C

YGK3

YGL088W

YGL152C

YGR001C

YGR017W

YGR054W

YGR114C

YGR115C

YGR206W

YGR266W

YHR029C

YHR162W

YIL130W

YIL137C

YIL157C

YIL172C

YIR030W-A

YIR042C

YJL010C

YJL018W

YJL097W

YJL175W

YJL178C

YJL192C

YJL218W

YJR015W

YJR030C

YKL044W

YKL097C

YKL132C

YKR040C

YKR045C

YKR064W

YLL055W

YLR021W

YLR065C

YLR124W

YLR143W

YLR187W

YLR428C

YMC1

YML040W

YMR099C

YMR103C

YMR166C

YMR244C-A

YMR245W

YMR252C

YMR269W

YMR318C

YNL057W

YNL058C

YNL083W

YNL168C

YNL190W

YNL310C

YNR009W

YOR029W

YOR086C

YOR286W

YOR331C

YPL158C

YPL201C

YPL208W

YPR050C

YPR091C

YPR098C

YRA1

YRA2

YRB1

ZDS2

Cluster8(node19)

Thereare389elements.

ADE3

ADE8

AFG1

AGE2

ALD6

AOS1

APL2

APL4

ARP4

ARP5

ARP9

ATP11

ATP15

AZR1

BBP1

BET1

BIG1

BIO3

BTS1

BUD2

CBP1

CBS2

CDC10

CDC25

CDC54

CET1

CHD1

CHL1

CHO1

CHS3

CKA1

CKA2

CKS1

CNM67

COF1

COT1

COX12

COX14

CPR8

CRD1

CSE2

CTK2

CUE4

CWH41

CWP1

CYK3

DAN2

DBF2

DCC1

DCP2

DDP1

DNM1

DSS4

EAF3

ECI1

ECM15

ECM18

ECM22

END3

EPS1

EST3

EXO70

EXO84

FAT1

FET3

FET5

FIP1

FLO8

FOL3

FSH1

GAL2

GAL80

GAS1

GGA2

GIS4

GPI16

GPM3

GRX5

GSG1

GTR1

GYP6

HBS1

HEX3

HOL1

HSC82

HSL1

HSN1

HST3

HTZ1

IDS2

INP53

IOC3

IRE1

IRR1

ISA1

ISA2

ISC1

IST2

IST3

KAR1

KEX1

KGD1

KIN2

KIN28

LAS21

LCB4

LEA1

LIP5

LIT1

LPD1

LRO1

LSB5

LSC1

LYS1

LYS5

MAF1

MAK32

MAM33

MAS2

MDM31

MEF1

MEP1

MET1

MET12

MGT1

MID1

MLH1

MLP2

MNT3

MNT4

MOD5

MRK1

MRP17

MRP4

MRP7

MRPL10

MRPL13

MRPL32

MRPS17

MRPS35

MSS51

NAM2

NAT2

NAT3

NCA2

NFS1

NHA1

NHX1

NIC96

NMA1

NNF2

NOT5

NST1

NUT2

OKP1

OPT1

ORC1

OSH3

OXA1

PAP1

PCL1

PCT1

PDR8

PEA2

PET123

PET309

PET8

PEX28

PEX5

PGD1

PHB2

PHO4

PHO80

PMD1

POS5

POX1

PPA2

PPN1

PRI1

PRI2

PSK2

PTA1

PTC1

PTC2

QNS1

RAD30

RAD5

RDS1

REB1

RGD2

RGM1

RGP1

RGR1

RHO2

RHO4

RIB5

RIO2

RLR1

RML2

RNR4

RNY1

ROT2

RPB4

RPO21

RRN10

RSM24

RSM27

RTG2

RTS1

SAC1

SAP190

SAS4

SAT4

SBA1

SCO1

SDC1

SDH3

SEC12

SEC20

SEC22

SED5

SET3

SIW14

SKT5

SLA2

SMD3

SNF1

SNF6

SNU114

SOL3

SPC72

SPT6

SRB7

SRP14

SSP1

STD1

STP1

SWI1

SWI6

TAF4

TCM62

TFA2

TFC1

THI6

TIM44

TLG2

TRK1

TRP4

TRR2

TVP38

UBP12

UGO1

UME1

USE1

VAC8

VPS33

VPS55

VPS61

VPS70

WHI2

XDJ1

YAL048C

YAL061W

YAR069C

YBL010C

YBL055C

YBR124W

YBR210W

YBR216C

YBR239C

YCK3

YCR026C

YCR049C

YDL073W

YDL203C

YDL237W

YDR010C

YDR031W

YDR079C-A

YDR084C

YDR090C

YDR199W

YDR215C

YDR266C

YDR442W

YDR458C

YDR459C

YDR541C

YEL016C

YEL030C-A

YEL043W

YEL068C

YER074W-A

YER076C

YER079C-A

YER080W

YER147C-A

YER152C

YER186C

YFL034W

YFYV12

YGL140C

YGL220W

YGR150C

YGR151C

YGR293C

YHC3

YHL041W

YHR033W

YHR034C

YHR083W

YHR115C

YHR182W

YIL024C

YIL088C

YIL102C

YIL151C

YJL022W

YJL064W

YJL118W

YJL162C

YJR012C

YJR024C

YJR061W

YJR142W

YKL053W

YKL137W

YKL169C

YKL207W

YKR022C

YKR074W

YKU70

YLL053C

YLL054C

YLR020C

YLR036C

YLR089C

YLR114C

YLR224W

YLR346C

YLR412W

YLR415C

YLR456W

YMD8

YML072C

YML081W

YML101C-A

YMR007W

YMR085W

YMR107W

YMR157C

YMR209C

YMR222C

YMR237W

YMR265C

YMR293C

YMR316C-B

YNL086W

YNL095C

YNL140C

YNL181W

YNL191W

YNL217W

YNL254C

YNL326C

YNR020C

YNR029C

YNR048W

YOR050C

YOR154W

YOR164C

YOR175C

YOR364W

YPL041C

YPL047W

YPL102C

YPL105C

YPL137C

YPL150W

YPL176C

YPR083W

YPR089W

YPR099C

YPR115W

YPR147C

YPS7

YPT52

YSY6

YUR1

Cluster9(node21)

Thereare402elements.

AAT2

ABF1

ACP1

ADH1

AEP2

AFT2

AGA1

AIP1

AIR1

ALG1

ALG11

ALG6

APN1

AQY2

ARF1

ARR1

ARV1

ASP1

ATP20

AVT1

AXL2

BCS1

BET3

BET5

BFA1

BRE2

BUB2

BUD16

CCT2

CCT5

CDC19

CDC50

CDC91

CDS1

CLG1

COD5

COG4

CPA2

CPR2

CSE1

CSM1

CSM3

CTM1

CTR2

DCW1

DIB1

DIE2

DNF1

DOP1

DOT1

DOT5

DPB3

DPM1

DSL1

DUO1

ECM31

ECM39

ECM40

ELG1

ENO2

EPT1

ERD1

ERG24

ERG4

ERP2

ERP5

ERR1

ERV41

ESP1

EST1

ETF-BETA

FAU1

FBA1

FYV9

FZO1

GAT3

GEA2

GIM5

GIN11

GMH1

GNA1

GNT1

GPI1

GPI11

GPI17

GPI2

GSF2

GSH2

GUP1

GVP36

HAP3

HCR1

HEM1

HFA1

HIR1

HIR2

HIS2

HOC1

HST4

HUA2

IBD2

ILM1

IMP1

INP51

KEM1

KRE29

KSS1

KTI12

LSM1

MAL33

MBA1

MCA1

MDR1

MEI5

MET18

MET22

MET32

MET8

MEX67

MKT1

MOG1

MON1

MON2

MRP2

MRP5

MRPL23

MRPL28

MRPL3

MRPL38

MRPL6

MRPL7

MRPL8

MRPS16

MRPS18

MRPS9

MRS2

MSC6

MSI1

MSM1

MSS116

MST1

NAS6

NAT5

NDC1

NGL1

NHP6A

NIF3

NMT1

NPC2

OCA1

OPI3

OST1

OST3

PAA1

PBP4

PCA1

PCL2

PCM1

PDA1

PDC5

PET56

PEX12

PFK2

PFS2

PFY1

PGM1

PHO86

PMI40

PMR1

PMT2

POB3

POP7

PRP38

PRP39

PRP8

PTC7

PUF3

PUF4

PUT2

QRI1

RAD23

RAD55

RAD9

REM50

RFA3

RFT1

RGA1

RMD9

RNA14

RNA15

RNH1

RNH70

RPB2

RRN11

RSA1

RSC2

RSM23

RTT102

RUB1

SBH1

SDT1

SEC1

SEC13

SEC21

SEC23

SEC27

SEC28

SEC61

SEH1

SEN2

SER1

SFT2

SHE3

SIN3

SIP3

SIR4

SLH1

SMC2

SMC4

SMD2

SMP3

SNO1

SNT309

SPC1

SPC97

SPC98

SPT4

SPT8

SRB8

SSH1

SSK2

SSS1

SVP26

SWD3

SWF5

TAF6

TAL1

TDH3

TEF1

TEF2

TFB4

TIM18

TOM37

TOM70

TOS2

TPT1

TRS120

TRS33

TUB2

UBC1

UBP14

URA4

URE2

URK1

UTP30

VAC14

VMA10

VMA5

VPS65

VPS66

VPS9

WBP1

WHI4

YAL037C-B

YAL044W-A

YAL047W-A

YAR023C

YBL104C

YBR096W

YBR141C

YBR206W

YBR209W

YBR219C

YBR220C

YBR232C

YBR255C-A

YBR261C

YBT1

YCL002C

YDJ1

YDL009C

YDL096C

YDL121C

YDL157C

YDL211C

YDL221W

YDR048C

YDR102C

YDR117C

YDR187C

YDR250C

YDR279W

YDR357C

YDR367W

YDR372C

YDR374C

YDR401W

YDR431W

YDR521W

YDR537C

YEL034C-A

YEN1

YER010C

YER064C

YER071C

YER140W

YFR007W

YFR024C

YFR043C

YGL050W

YGL060W

YGL069C

YGL132W

YGL204C

YGR033C

YGR058W

YGR257C

YHL005C

YHL012W

YHR078W

YHR122W

YHR145C

YHR173C

YHR186C

YIL039W

YIL083C

YIL103W

YIP1

YIR010W

YJL065C

YJL072C

YJL120W

YJL181W

YJR097W

YJR136C

YJR141W

YKL063C

YKL084W

YKL161C

YKL177W

YKR005C

YKR027W

YLL030C

YLL032C

YLL047W

YLL049W

YLR007W

YLR031W

YLR053C

YLR063W

YLR111W

YLR253W

YLR324W

YLR326W

YLR365W

YLR385C

YLR424W

YLR426W

YML030W

YML079W

YML094C-A

YML108W

YML119W

YML122C

YMR003W

YMR098C

YMR118C

YMR163C

YMR185W

YMR226C

YMR299C

YND1

YNL022C

YNL080C

YNL108C

YNL129W

YNL198C

YNL275W

YNL320W

YNR063W

YNR064C

YNT20

YOL026C

YOL027C

YOL107W

YOL134C

YOL146W

YOL157C

YOR008C-A

YOR015W

YOR062C

YOR102W

YOR118W

YOR203W

YOR251C

YOR262W

YOR352W

YPL066W

YPL267W

YPR053C

YPT32

YPT7

Cluster10(node22)

Thereare311elements.

ADE1

ADE13

ADE4

ADH2

ADO1

ALG5

ALG8

APG10

APJ1

ARC1

ARL1

ARO2

ARO3

ASN2

ATC1

AUR1

BAT2

BCP1

BGL2

BIM1

BPL1

BUD17

CAF16

CBC2

CBR1

CBS1

CCT3

CCT4

CCT6

CCT7

CCT8

CDC36

CIS3

CLU1

COG6

COP1

CPR7

CSN9

CSR1

CTP1

DBR1

DCG1

DCP3

DEG1

DIM1

DLT1

DOM34

DPH5

DRE3

DUS1

ECM1

EFR3

ELP6

ERG13

ERG7

ERV1

ERV46

FAP7

FEN1

FRE1

GBP2

GCD1

GCD14

GCD6

GLE2

GSP1

GUK1

HAM1

HEM2

HIF1

HIS1

HKR1

HMF1

HOM6

HPA3

HPT1

HRP1

HXK2

IDP1

IDP3

IKI1

ILV1

IMG1

ISW1

ITR2

KAP114

KRE26

KRE27

KRE30

KTR3

LHP1

LOT5

LSM4

LSM5

LSM6

LST4

LYP1

MAK10

MCD1

MCM6

MDL1

MDM20

MET17

MEU1

MGE1

MHT1

MIR1

MKC7

MNN10

MNN5

MNN9

MRF1

MRP8

MRPL20

MRT4

NCE101

NIS1

NIT3

NSP1

NTF2

NTO1

NUP100

NUP157

NUP170

NUP60

ODC2

ORT1

OST5

OST6

PAB1

PDB1

PER1

PFK1

PGI1

PHO88

PKR1

POL30

POL32

POM152

POP4

PPE1

PPR1

PRO2

PRP16

PRY2

PRY3

PSH1

PTP3

PUF6

RAD27

RAM2

RAS1

RAX2

RCE1

REX4

RFC2

RFC5

RNH35

RPC10

RPC25

RPC31

RPR46

RRI1

RRP4

RRP40

RRP45

RSM26

RTN1

SAD1

SAS2

SBP1

SCY1

SEC53

SEC65

SEC7

SHR3

SIT1

SKI3

SMB1

SOF1

SPB4

SPC3

SQT1

SRP1

SRP21

SRP68

SSL1

STE23

SUB2

SUC2

SUI3

SUP45

SUR4

SVF1

SYP1

TAF13

TBF1

TCP1

THR1

THR4

TIM8

TOM1

TOM22

TOM71

TOS7

TRM2

TRM5

TRM9

TRP2

TRP5

TSC13

TUB3

URA2

URA6

URM1

VGR4

VMA22

VMA6

VTS1

WTM2

YAH1

YAL045C

YBL009W

YBR012C

YBR032W

YBR042C

YBR089W

YBR277C

YDL016C

YDL201W

YDR094W

YDR114C

YDR115W

YDR154C

YDR514C

YEL001C

YEL045C

YER156C

YFR011C

YFR038W

YFR044C

YGL239C

YGR024C

YGR073C

YGR079W

YGR093W

YGR190C

YGR265W

YGR283C

YHL026C

YHR069C-A

YHR111W

YHR133C

YIL096C

YIL110W

YJL051W

YJL122W

YJL182C

YJL193W

YJL206C-A

YJR111C

YKL047W

YKL056C

YKL115C

YKL118W

YKR012C

YKR047W

YKR065C

YLL014W

YLR008C

YLR042C

YLR049C

YLR236C

YLR287C

YMC2

YML084W

YML125C

YMR130W

YMR221C

YMR259C

YMR321C

YNL010W

YNL122C

YNL136W

YNL152W

YNL162W-A

YNL203C

YNL234W

YNL313C

YNR042W

YOL007C

YOL070C

YOL075C

YOL079W

YOR051C

YOR093C

YOR165W

YOR282W

YOR283W

YOR345C

YOR390W

YPL034W

YPL062W

YPL238C

YPL251W

YPL264C

YPL279C

YPR109W

YPR170C

YPT31

YVH1

Cluster11(node8)

Thereare1761elements.

AAD10

AAD15

AAD16

AAD3

AAD4

AAD6

AAT1

ABC1

ABD1

ABF2

ABM1

ABP1

ABP140

ABZ1

ACA1

ACB1

ACN9

ACS1

ADE16

ADH5

ADH7

ADP1

ADR1

ADY2

ADY4

AEP1

AFG3

AFR1

AGP2

AGX1

AHT1

AIR2

AKL1

AKR1

AKR2

ALG2

ALG7

ALR2

AMD2

AME1

AMS1

ANB1

ANT1

AOR1

APA1

APC1

APC11

APC2

APC4

APG1

APG12

APG13

APG17

APL1

APL5

APN2

APP1

APS1

APS3

ARC18

ARC35

ARF2

ARG2

ARG3

ARG4

"ARG5_6"

ARG82

ARH1

ARL3

ARN1

ARN2

ARO10

ARP10

ARP3

ASF2

ASG7

ASI1

ASK10

ASP3

ATE1

ATF1

ATG21

ATP10

ATP4

ATP7

ATX1

AUA1

AUS1

AUT2

AUT4

AVO2

AVT3

BCK1

BDF2

BEM2

BEM3

BEM4

BET2

BET4

BFR1

BIK1

BIO4

BIO5

BIR1

BIT1

BLM3

BMH2

BNA1

BNA2

BNA3

BNA4

BNA5

BNA6

BNI1

BNR1

BOP2

BOS1

BPT1

BRE5

BUB3

BUD5

BUD7

BUL1

BUR2

BYE1

CAF17

CAK1

CAP1

CAP2

CAT2

CAT5

CAT8

CBK1

CBP2

CBP3

CBP6

CBT1

CCC2

CCH1

CCL1

CCZ1

CDC11

CDC13

CDC15

CDC16

CDC2

CDC21

CDC24

CDC28

CDC37

CDC4

CDC40

CDC45

CDC46

CDC48

CDC53

CDC6

CDC73

CDC8

CDC9

CEG1

CEM1

CFT1

CFT18

CHA4

CHS1

CIK1

CIN4

CIT1

CKI1

CLB4

CLP1

CMD1

CMP2

CNB1

COG1

COG2

COG5

COQ1

COQ4

COQ5

COQ6

COS111

COX11

COX15

COX16

COX17

COX20

COX8

COY1

CPD1

CPR4

CPR5

CPS1

CRC1

CRH1

CRM1

CRN1

CRR1

CRZ1

CSG2

CSI1

CSM2

CSN12

CST9

CTF13

CTF3

CTK1

CTK3

CTS2

CUE1

CUE2

CUS2

CVT17

CVT19

CVT20

CVT9

CWC23

CYB5

CYC1

CYC2

CYR1

CYT2

DAD4

DAK1

DAK2

DAL4

DAL82

DAP2

DBP1

DCP1

DCS1

DCS2

DDC1

DDI1

DDR48

DED1

DEF1

DEM1

DER1

DFG10

DFG16

DFR1

DGA1

DIA2

DIA3

DIG2

DIT2

DLD1

DMC1

DNF3

DNL4

DOA1

DOA4

DOG1

DOG2

DPB2

DPP1

DST1

DUN1

"DUR1_2"

ECM10

ECM19

ECM2

ECM21

ECM27

ECM32

ECM37

ECM38

ECM4

ECM7

EDE1

EDS1

ELM1

ELP4

EMI1

EMP24

EMP46

EMP47

ENO1

ENT4

ERD2

ERF2

ERG27

ERG28

ERG8

ERO1

ERP1

ERP6

ERR2

ERV14

ERV2

ERV25

ERV29

ESBP6

ESC1

ESC2

EUG1

EXG2

FAA2

FAD1

FAP1

FAR3

FAR8

FBP26

FDH1

FEN2

FIG4

FIL1

FMN1

FMS1

FMT1

FPR2

FRE6

FTH1

FUN31

FUS1

FYV4

FYV6

FZF1

GAA1

GAL10

GAL11

GAL3

GAL83

GCD10

GCN2

GCR1

GCV3

GCY1

GDH3

GDI1

GFA1

GIF1

GIT1

GLC3

GLC7

GLK1

GLN3

GLO1

GLO2

GND2

GON3

GPB1

GPI10

GPI12

GPI13

GPI8

GPM1

GPM2

GPT2

GRC3

GRE2

GRH1

GRR1

GRX2

GRX3

GSC2

GSP2

GTR2

GTT1

GTT3

GUF1

GYP1

HAA1

HAC1

HAL5

HAL9

HAT1

HBT1

HCH1

HCS1

HEM14

HEM15

HIS4

HIS5

HIT1

HLJ1

HLR1

HMI1

HMRA2

HNT1

HNT3

HOG1

HOP1

HOR2

HOS1

HOS2

HPA2

HPC2

HPR5

HRB1

HRD1

HRD3

HRT1

HRT2

HSD1

HSF1

HSH155

HSH49

HSM3

HSP10

HSP12

HSP150

HSP26

HSP82

HST1

HST2

HTL1

HUL4

HUL5

HUT1

HXT10

HXT11

HXT13

HXT15

HXT16

HXT17

HXT5

HXT9

IAH1

ICL1

ICL2

ICY1

IES3

IES4

IES5

IFM1

IKS1

IMG2

IML1

IML3

INH1

INO1

INO4

INO80

IOC2

IOC4

IPK1

IPL1

IRA1

IRA2

IRS4

ISW2

ISY1

ITC1

IWR1

JAC1

JEM1

JEN1

JIP3

JSN1

KAP122

KAP95

KAR2

KAR4

KAR9

KEL1

KEL2

KIN1

KIP3

KKQ8

KRE25

KRE28

KRE5

KTR1

KTR2

KTR4

KTR5

KTR6

LAP3

LCD1

LDB7

LEE1

LGE1

LHS1

LIF1

LIN1

LIP2

LOT6

LPP1

LRG1

LRS4

LSC2

LSM8

LST7

LST8

LTP1

LUV1

LYS7

LYS9

MAC1

MAD2

MAG1

MAG2

MAL12

MAM3

MAS1

MATALPHA2

MBB1

MBF1

MCH1

MCH4

MCM16

MCM21

MCM22

MCT1

MDH3

MDJ2

MDL2

MDM10

MDM12

MDS3

MEC1

MED11

MED4

MEI4

MEK1

MER1

MET10

MET14

MET16

MET30

MET4

MGS1

MHP1

MIA40

MIF2

MIH1

MIP6

MKK2

MLH2

MLS1

MMP1

MMS1

MMS21

MMS22

MMT2

MNE1

MNN4

MNR2

MOH1

MOR1

MOT2

MPA43

MPC54

MPD1

MPD2

MPE1

MPH1

MPH2

MPS2

MPT5

MRH4

MRLP39

MRM2

MRP10

MRP49

MRPL17

MRPL19

MRPL22

MRPL33

MRPL36

MRPL44

MRPL50

MRS5

MSB3

MSB4

MSE1

MSH2

MSH4

MSK1

MSL1

MSN1

MSN5

MSP1

MSR1

MSS1

MSS2

MSS4

MST27

MST28

MSU1

MSW1

MSY1

MTD1

MTF1

MTF2

MTW1

MUB1

MUD2

MUS81

MVP1

MYO3

MYO4

NAB2

NAB3

NAM9

NAR1

NBP1

NBP35

NCE103

NCE4

NEO1

NFI1

NGR1

NHP10

NHP6B

NIP100

NMA2

NOP12

NOP13

NPL3

NPL4

NPR1

NPR2

NTA1

NTG1

NTH2

NUP116

NUP188

NUP49

NUP53

NYV1

OAF1

OCH1

OPY1

ORC3

ORM1

OSH2

OSH6

OST2

OSW1

OTCL1

OYE2

OYE3

PAC2

PAD1

PAF1

PAI3

PAK1

PAM1

PAN2

PAN3

PAU2

PAU3

PAU4

PAU5

PAU6

PAU7

PBN1

PBP2

PBS2

PCF11

PCH2

PCK1

PCL10

PCL5

PCP1

PDE1

PDE2

PDI1

PDR1

PDR10

PDR11

PDR3

PDX1

PDX3

PEP1

PEP5

PEP7

PEP8

PES4

PET100

PET111

PET18

PET191

PET54

PEX1

PEX10

PEX13

PEX14

PEX15

PEX17

PEX3

PEX4

PEX8

PGK1

PGS1

PGU1

PHA2

PHM7

PHO13

PIB2

PIG1

PIG2

PIK1

PIM1

PIN2

PIP2

PIR1

PIR3

PKH1

PKH2

PLB1

PLC1

PLO2

PLP1

PMC1

PMS1

PMT1

PMT3

PMT5

PMU1

POL4

POP1

PPG1

PPH3

PPQ1

PPZ1

PRC1

PRD1

PRE1

PRE10

PRE2

PRE3

PRE4

PRE5

PRE6

PRE7

PRE8

PRE9

PRK1

PRM1

PRM2

PRM3

PRM8

PRM9

PRP12

PRP2

PRP21

PRP22

PRP4

PRP40

PRP42

PRP46

PRP5

PRY1

PSA1

PSD1

PSD2

pseudo-CPS1-hom

PSY3

PTC3

PTC5

PTH1

PTI1

PTK2

PTM1

PTP2

PTR3

PUB1

PUP1

PUP2

PUP3

PUT3

PYC1

PYC2

PYK2

QRI2

QRI8

RAD14

RAD16

RAD2

RAD24

RAD52

RAD53

RAD54

RAD57

RAD6

RAD7

RAS2

RAV1

RAV2

RBK1

RBL2

RCK1

RCK2

RDS2

REC102

REG1

REG2

RER2

RET2

RET3

REX3

RFA1

RFA2

RFX1

RGD1

RGT1

RHC18

RIB1

RIB7

RIC1

RIM101

RIM15

RIM20

RIM21

RIM8

RIM9

RIS1

RIT1

RMD11

RMD6

RMS1

RNH203

RNR2

RNR3

ROG1

ROM1

ROM2

ROX3

RPA49

RPB11

RPC17

RPD3

RPM2

RPN1

RPN11

RPN12

RPN13

RPN14

RPN2

RPN3

RPN4

RPN5

RPN6

RPN7

RPN8

RPN9

RPO41

RPS29A

RPT1

RPT2

RPT4

RPT6

RRD2

RRI2

RRM3

RRP43

RSC1

RSC3

RSC30

RSC4

RSC58

RSC9

RSE1

RSM18

RSM19

RTA1

RTT101

RTT105

RTT106

RXT2

SAC7

SAE2

SAP155

SAP4

SAR1

SAS5

SAT2

SCC2

SCL1

SCO2

SCP1

SCS3

SCT1

SDC25

SDH4

SEC10

SEC11

SEC15

SEC16

SEC17

SEC2

SEC24

SEC3

SEC34

SEC5

SEC59

SEC6

SEC62

SEC63

SEC66

SEC9

SED4

SEF1

SEM1

SEN54

SER2

SER3

SER33

SET2

SFA1

SFB2

SFB3

SFI1

SGE1

SGF29

SGT1

SGT2

SHC1

SHE10

SHE2

SHE4

SHG1

SHM2

SHP1

SHU2

SIP4

SIR1

SIZ1

SKI2

SKI7

SLA1

SLC1

SLD2

SLD3

SLK19

SLT2

SLX1

SLY1

SLZ1

SMA1

SMA2

SMC3

SMF3

SMK1

SML1

SMT3

SMX3

SMY1

SNC1

SNF3

SNF8

SNM1

SNO2

SNO3

SNO4

SNU23

SNZ1

SNZ2

SNZ3

SOH1

SOK1

SOL2

SOR1

SOR2

SPC105

SPC19

SPC2

SPC34

SPO1

SPO11

SPO13

SPO20

SPO21

SPO22

SPO69

SPO7

SPO71

SPR1

SPR28

SPR3

SPS1

SPS18

SPS19

SPT2

SPT23

SPT3

SPT7

SRB4

SRB6

SRC1

SRI1

SRN2

SRO77

SRY1

SSA3

SSA4

SSC1

SSD1

SSE1

SSE2

SSH4

SSM4

SSP120

SSP2

SSQ1

SSU1

SSU72

SSY1

STB6

STE11

STE14

STE18

STE24

STE5

STE7

STF1

STL1

STN1

STP3

STR3

STS1

SUA7

SUE1

SUI1

SUL2

SUN4

SWA2

SWD1

SWF1

SWI4

SWM1

SWP1

SWR1

SWS2

SYM1

SYN8

SYS1

SYT1

TAD2

TAF1

TAF10

TAF3

TAF67

TAH11

TAO3

TAP42

TBS1

TCM10

TDH1

TDH2

TFC3

TFC4

TFC6

TFC7

TFC8

TGL1

TGL3

THI12

THI13

THI2

THI20

THI21

THI5

THI7

THO1

TIF4631

TIM50

TKL2

TLG1

TMS1

TMT1

TOA1

TOA2

TOM5

TOP1

TOP3

TOR1

TOR2

TOS8

TPM1

TPP1

TPS1

TRK2

TRS130

TRX2

TRX3

TSA1

TSL1

TUL1

TUS1

TYR1

UBA1

UBC5

UBC8

UBI4

UBP11

UBP15

UBP16

UBP2

UBP6

UBP9

UBR1

UBR2

UBS1

UFD1

UFD2

UFD4

UFE1

UFO1

UGA1

UGP1

ULA1

ULP2

UMP1

UNG1

URH1

USA1

UTR4

VAC7

VID21

VID22

VID24

VID27

VID30

VPH2

VPS13

VPS15

VPS20

VPS25

VPS34

VPS35

VPS36

VPS38

VPS41

VPS45

VPS5

VPS60

VPS63

VPS64

VPS67

VPS68

VPS73

VPS8

VTH1

WAR1

WSS1

XPT1

XRS2

XYL2

YAF9

YAK1

YAL027W

YAL046C

YAL053W

YAL064W-B

YAP1

YAP5

YAP6

YAR030C

YAR1

YAT1

YBL012C

YBL065W

YBL073W

YBL107C

YBR007C

YBR014C

YBR022W

YBR051W

YBR052C

YBR053C

YBR063C

YBR064W

YBR071W

YBR077C

YBR116C

YBR144C

YBR147W

YBR168W

YBR197C

YBR204C

YBR241C

YBR246W

YBR280C

YBR284W

YBR285W

YBR293W

YCG1

YCL003W

YCL020W

YCL021W-A

YCL026C-B

YCL044C

YCL047C

YCL048W

YCL049C

YCL056C

YCL069W

YCL073C

YCL075W

YCR007C

YCR013C

YCR023C

YCR024C

YCR082W

YCR100C

YDL010W

YDL011C

YDL012C

YDL025C

YDL026W

YDL027C

YDL036C

YDL057W

YDL068W

YDL085C-A

YDL089W

YDL091C

YDL094C

YDL099W

YDL100C

YDL118W

YDL119C

YDL129W

YDL144C

YDL146W

YDL158C

YDL162C

YDL172C

YDL173W

YDL177C

YDL183C

YDL193W

YDL196W

YDL199C

YDL206W

YDL233W

YDL238C

YDR003W

YDR008C

YDR015C

YDR026C

YDR029W

YDR034W-B

YDR065W

YDR067C

YDR089W

YDR107C

YDR109C

YDR128W

YDR131C

YDR132C

YDR149C

YDR163W

YDR185C

YDR203W

YDR220C

YDR221W

YDR249C

YDR262W

YDR269C

YDR282C

YDR287W

YDR307W

YDR314C

YDR316W

YDR327W

YDR332W

YDR333C

YDR336W

YDR338C

YDR355C

YDR366C

YDR379C-A

YDR411C

YDR474C

YDR476C

YDR479C

YDR489W

YDR520C

YDR531W

YDR535C

YDR539W

YDR540C

YEA4

YEL010W

YEL020C

YEL041W

YEL047C

YEL059W

YEL064C

YEL067C

YEL073C

YER004W

YER030W

YER034W

YER039C-A

YER051W

YER066W

YER077C

YER084W-A

YER093C

YER093C-A

YER097W

YER128W

YER135C

YER138C

YER139C

YER160C

YER163C

YER181C

YER182W

YET1

YFH1

YFL006W

YFL032W

YFL042C

YFL044C

YFL046W

YFL049W

YFL061W

YFR003C

YFR008W

YFR020W

YFR022W

YFR026C

YFR039C

YFR041C

YFR042W

YFR045W

YFR046C

YGL010W

YGL042C

YGL046W

YGL047W

YGL052W

YGL057C

YGL059W

YGL068W

YGL074C

YGL080W

YGL081W

YGL082W

YGL117W

YGL131C

YGL149W

YGL157W

YGL160W

YGL168W

YGL176C

YGL177W

YGL179C

YGL185C

YGL231C

YGL242C

YGL262W

YGR002C

YGR003W

YGR012W

YGR026W

YGR039W

YGR043C

YGR045C

YGR053C

YGR067C

YGR110W

YGR117C

YGR122W

YGR126W

YGR182C

YGR198W

YGR201C

YGR210C

YGR212W

YGR223C

YGR250C

YGR263C

YGR277C

YGR287C

YGR291C

YHL034W-A

YHR022C

YHR035W

YHR045W

YHR054C

YHR067W

YHR080C

YHR100C

YHR112C

YHR113W

YHR116W

YHR139C-A

YHR140W

YHR151C

YSP1

YHR159W

YHR180W

YHR209W

YIF1

YIL001W

YIL023C

YIL029C

YIL040W

YIL067C

YIL082W

YIL105C

YIL108W

YIL135C

YIL141W

YIL142C-A

YIL152W

YIL166C

YIL174W

YIL176C

YIM1

YIP3

YIR007W

YIR041W

YIR043C

YIR044C

YJL015C

YJL016W

YJL017W

YJL021C

YJL027C

YJL032W

YJL038C

YJL046W

YJL049W

YJL062W-A

YJL068C

YJL091C

YJL119C

YJL132W

YJL147C

YJL150W

FMP33

YJL163C

YJL169W

YJL195C

YJL206C

YJL220W

YJR011C

YJR023C

YJR027W

YJR028W

YJR029W

YJR037W

YJR039W

YJR054W

YJR079W

YJR080C

YJR107W

YJR116W

YJR119C

YJR157W

YJR160C

YKE2

YKL036C

YKL061W

YKL066W

YKL071W

YKL091C

YKL098W

YKL102C

YKL123W

YKL147C

YKL162C-A

YKL174C

YKL189W

YKL206C

YKL215C

YKL222C

YKL224C

YKR015C

YKR017C

YKR018C

YKR021W

YKR032W

YKR033C

YKR035C

YKR041W

YKR051W

YKR070W

YKR073C

YKR096W

YKT6

YKT9

YKU80

YLF2

YLL017W

YLL023C

YLL025W

YLL029W

YLL033W

YLL057C

YLL058W

YLL059C

YLL064C

YLR004C

YLR023C

YLR040C

YLR041W

YLR046C

YLR047C

YLR050C

YLR057W

YLR064W

YLR091W

YLR101C

YLR104W

YLR108C

YLR112W

YLR118C

YLR126C

YLR128W

YLR132C

YLR156W

YLR164W

YLR169W

YLR171W

YLR173W

YLR194C

YLR199C

YLR201C

YLR202C

YLR217W

YLR225C

YLR230W

YLR232W

YLR235C

YLR241W

YLR247C

YLR252W

YLR269C

YLR278C

YLR280C

YLR281C

YLR282C

YLR290C

YLR294C

YLR296W

YLR301W

YLR302C

YLR311C

YLR323C

YLR327C

YLR334C

YLR345W

YLR356W

YLR364W

YLR366W

YLR379W

YLR387C

YLR408C

YLR422W

YLR431C

YLR434C

YLR444C

YLR454W

YLR458W

YLR460C

YME1

YML002W

YML005W

YML011C

YML034C-A

YML037C

YML041C

YML050W

YML057C-A

YML059C

YML096W

YML131W

YMR010W

YMR031W-A

YMR034C

YMR040W

YMR041C

YMR057C

YMR067C

YMR073C

YMR074C

YMR082C

YMR086C-A

YMR1

YMR115W

YMR119W-A

YMR122C

YMR158W

YMR160W

YMR178W

YMR181C

YMR184W

YMR187C

YMR191W

YMR194C-A

YMR204C

YMR210W

YMR211W

YMR31

YMR315W

YMR323W

YNK1

YNL011C

YNL040W

YNL043C

YNL045W

YNL046W

YNL063W

YNL089C

YNL092W

YNL105W

YNL109W

YNL115C

YNL116W

YNL119W

YNL127W

YNL134C

YNL146W

YNL149C

YNL150W

YNL155W

YNL156C

YNL157W

YNL158W

YNL165W

YNL171C

YNL179C

YNL193W

YNL200C

YNL205C

YNL211C

YNL213C

YNL226W

YNL235C

YNL260C

YNL276C

YNL285W

YNL324W

YNL335W

YNR036C

YNR040W

YNR047W

YNR061C

YNR062C

YNR068C

YNR074C

YOL015W

YOL035C

YOL036W

YOL037C

YOL046C

YOL048C

YOL053W

YOL073C

YOL078W

YOL087C

YOL099C

YOL106W

YOL159C

YOL161C

YOL162W

YOL163W

YOL164W

YOR019W

YOR022C

YOR024W

YOR059C

YOR068C

YOR071C

YOR097C

YOR1

YOR105W

YOR111W

YOR131C

YOR138C

YOR152C

YOR172W

YOR192C

YOR193W

YOR199W

YOR200W

YOR223W

YOR227W

YOR228C

YOR256C

YOR285W

YOR289W

YOR318C

YOR322C

YOR324C

YOR325W

YOR338W

YOR343C

YOR356W

YOR378W

YOR379C

YOR380W

YOR389W

YOR394W

YPC1

YPD1

YPK1

YPL005W

YPL033C

YPL035C

YPL039W

YPL052W

YPL067C

YPL088W

YPL099C

YPL103C

YPL109C

YPL110C

YPL113C

YPL136W

YPL141C

YPL162C

YPL168W

YPL184C

YPL191C

YPL196W

YPL225W

YPL257W

YPL260W

YPL272C

YPR003C

YPR011C

YPR012W

YPR039W

YPR076W

YPR078C

YPR085C

YPR117W

YPR125W

YPR126C

YPR130C

YPR150W

YPR153W

YPR172W

YPR174C

YPR177C

YPR195C

YPR197C

YPS1

YPS3

YPS5

YPS6

YPT1

YRB2

YRB30

YRR1

YSN1

YSP3

YSR3

YTP1

YUH1

YVC1

ZAP1

ZIP2

ZRG17

ZRG8

ZRT3

ZTA1

ZWF1

Cluster12(node23)

Thereare718elements.

AAR2

ACF2

ADE12

ADE17

ADE2

ADH4

ADY3

AGP3

AHC1

AHP1

ALD2

ALD4

ALP1

APC9

APG14

APG16

APG5

APG7

APG9

APM4

APS2

AQR2

ARA1

ARO80

ARO9

ASF1

ASI2

ASI3

ASM4

AST2

ATH1

ATR1

AUT1

AUT10

AUT7

AVT6

AYR1

BBC1

BCY1

BDH1

BOP3

BSC3

BSC4

BSD2

BST1

BUD13

BUD3

BUR6

CAC2

CAD1

CAF4

CAN1

CAX4

CCP1

CDC23

CDC31

CDC34

CDC55

CDH1

CHK1

CHS5

CHS6

CIS1

CLB3

CLC1

CLF1

CMK1

COQ3

COX4

COX6

CPR6

CRP1

CST6

CTA1

CTF8

CTH1

CTL1

CUE3

CUP2

CWC22

CYB2

CYC7

DAL80

DAN3

DCR1

DCR2

DIA1

DIG1

DIT1

DLD2

DON1

DOS2

DTD1

DUR3

DYN2

EAF5

EAF6

EAP1

ECM25

ECM3

ECM30

ECO1

ELP2

EMI2

EMI5

ENA2

ENT2

ETR1

FAB1

FCY22

FDH2

FES1

FIN1

FIS1

FIT1

FIT3

FLO1

FMC1

FMO

FOB1

FRE3

FRE5

FRQ1

FRT2

FSP2

FUN14

FUN21

FUN34

FUN57

FUS2

FYV10

GAD1

GAL1

GAT4

GCV1

GDB1

GEF1

GGA1

GIP1

GIP2

GIS1

GLC8

GLG2

GPD1

GPR1

GRD19

GRE3

GRX1

GSY1

GTT2

GUT2

GYP7

HAL1

HAP1

HAT2

HEM13

HMS1

HMX1

HOR7

HOS4

HRT3

HSP30

HSP42

HSP60

HSP78

HXK1

HXT8

HYR1

HYS2

ICS2

ICT1

IDP2

IES6

IME1

IME2

IME4

IML2

IMP2

INP54

IST1

ITT1

IXR1

KHA1

KIN82

KNS1

KRE1

KRE22

LAG2

LAP4

LCB5

LRE1

LSB6

LSP1

LUC7

MAD1

MAD3

MAL13

MCM3

MCR1

MDG1

MDJ1

MDM34

MEP2

MET13

MET28

MET3

MGA1

MKS1

MMF1

MMS4

MND1

MND2

MNS1

MOH2

MOT3

MPM1

MPS3

MRC1

MRP21

MRP51

MRPL1

MRPL16

MRPL25

MRPS28

MRS4

MSC1

MSC3

MSN2

MTC2

MTL1

MTR2

MUP3

NAB6

NBP2

NCA3

NDE2

NDI1

NEM1

NFU1

NGG1

NGL3

NMD4

NRG1

NTG2

NUM1

NUT1

OM45

OPY2

ORC5

PAU1

PCI8

PCL8

PDC6

PDS1

PEP12

PEP4

PET112

PET117

PET130

PEX18

PEX19

PFK26

PGM2

PHM8

PHO2

PHO23

PHO36

PHR1

PIB1

PIN3

PLM2

PMP2

PNC1

PNG1

POP3

POR1

POT1

PPH22

PPT2

PRB1

PRM4

PRM5

PRX1

PSO2

PST2

PTP1

PUS5

PUT1

PUT4

PXA2

QCR6

QCR7

QCR8

QCR9

QDR1

RAD10

RAD17

RAD18

RAD4

RAD51

RCN1

RDS3

REC104

REC114

RHO5

RIM11

RIM13

RIM4

RLM1

RMD8

ROT1

RPH1

RPR2

RRD1

RSB1

RSM22

RSM28

RTG3

RTN2

RTT103

RVS167

RXT3

SAC6

SAP1

SCJ1

SCM3

SDH1

SDH2

SDS22

SDS24

SED1

SET4

SHY1

SIA1

SIP2

SIP5

SIS1

SIS2

SKM1

SKO1

SKP1

SMC1

SMF1

SNA4

SNF2

SNF7

SNG1

SNT1

SNU71

SOD2

SOL1

SOL4

SPC110

SPC29

SPH1

SPI1

SPO74

SPO77

SPP381

SPS100

SPT20

SSA1

SSA2

SSK1

SSN8

SSO1

STB2

STB3

STB5

STE13

STF2

STI1

STR2

SUB1

SUL1

SWC1

TDP1

TEP1

TFB1

TFS1

TGL2

THI3

TIM11

TIP1

TIS11

TOS5

TPK1

TPK2

TPN1

TPO1

TPO4

TPS2

TRP1

TSA2

TVP15

TWF1

UBC13

UBC4

UBP5

UGA4

UIP3

ULP1

UME6

URA8

UTR1

VAB2

VEL1

VHS3

VPS16

VPS21

VPS27

VPS3

VPS30

VPS62

VPS72

VTH2

WHI3

WTM1

XBP1

YAL004W

YAL049C

YAL065C

YAP1801

YAR062W

YBL046W

YBL059W

YBL094C

YBR004C

YBR016W

YBR027C

YBR047W

YBR056W

YBR062C

YBR108W

YBR113W

YBR161W

YBR184W

YBR225W

YBR230C

YBR235W

YBR250W

YBR255W

YBR287W

YCF1

YCL033C

YCR006C

YCR025C

YCR050C

YCR051W

YCR061W

YCR099C

YCR101C

YCS4

YDC1

YDL034W

YDL071C

YDL072C

YDL086W

YDL124W

YDL156W

YDL163W

YDL180W

YDL218W

FMP45

YDR018C

YDR056C

YDR061W

YDR124W

YDR196C

YDR209C

YDR210W

YDR214W

YDR222W

YDR223W

YDR230W

YDR248C

YDR271C

YDR291W

YDR306C

YDR319C

YDR330W

YDR340W

YDR344C

YDR348C

YDR387C

YDR415C

YDR428C

YDR455C

YDR506C

YDR533C

YEL023C

YEL057C

YER053C

YER067W

YER079W

YER084W

YER087W

YER130C

YER158C

YER188W

YFL015W-A

YFL043C

YFL051C

YFL052W

YFL054C

YFR012W

YFR016C

YFR017C

YFR035C

YFR054C

YGL072C

YGL079W

YGL101W

YGL165C

YGL196W

YGL217C

YGL230C

YGL250W

YGL261C

YGP1

YGR016W

YGR022C

YGR051C

YGR106C

YGR111W

YGR125W

YGR127W

YGR176W

YGR205W

YGR226C

YGR237C

YGR294W

YHL008C

YHL021C

YHL046C

YHR048W

YHR087W

YHR097C

YHR126C

YHR138C

YHR202W

YIL006W

YIL007C

YIL055C

YIL056W

YIL058W

YIL077C

YIL087C

YIL089W

YIL113W

YIL121W

YIR003W

YIR014W

YJL045W

YJL048C

YJL055W

YJL067W

YJL083W

YJL103C

YJL114W

YJL142C

YJL149W

YJL152W

YJL160C

YJL171C

YJL211C

YJL213W

YJL216C

YJL217W

YJR008W

YJR038C

YJR096W

YJR098C

YJR128W

YJR149W

YJR154W

YJU3

YKL031W

YKL037W

YKL070W

YKL100C

YKL107W

YKL121W

YKL133C

YKL151C

YKL162C

YKL171W

YKL187C

YKR007W

YKR016W

YKR023W

YKR046C

YKR049C

YKR078W

YKR087C

YKR105C

YLL007C

YLL020C

YLL037W

YLL056C

YLR001C

YLR054C

YLR137W

YLR140W

YLR149C

YLR177W

YLR184W

YLR218C

YLR238W

YLR254C

YLR255C

YLR257W

YLR271W

YLR283W

YLR312C

YLR374C

YLR392C

YLR414C

YLR437C

YLR445W

YLR455W

YML013W

YML020W

YML083C

YML089C

YML116W-A

YMR002W

YMR009W

YMR018W

YMR030W

YMR071C

YMR084W

YMR087W

YMR090W

YMR110C

YMR111C

YMR135W-A

YMR141C

YMR155W

YMR206W

YMR251W

YMR253C

YMR254C

YMR258C

YMR262W

YMR291W

YMR298W

YMR316C-A

YMR317W

YMR325W

YNL013C

YNL018C

YNL019C

YNL026W

YNL033W

YNL056W

YNL100W

YNL123W

YNL194C

YNL195C

YNL208W

YNL224C

YNL253W

YNL266W

YNL274C

YNL305C

YNR005C

YNR014W

YNR021W

YNR069C

YOL008W

YOL032W

YOL083W

YOL085C

YOR012W

YOR042W

YOR044W

YOR052C

YOR055W

YOR060C

YOR082C

YOR161C

YOR220W

YOR268C

YOR292C

YOR304C-A

YOR385W

YOR387C

YOS9

YPL009C

YPL077C

YPL107W

YPL159C

YPL180W

YPL183W-A

YPL222W

YPL236C

YPL245W

YPL247C

YPL280W

YPR022C

YPR061C

YPR081C

YPR140W

YPR146C

YPR158W

YPT10

YPT11

YPT35

YPT53

YPT6

YSC84

YSW1

YTA7

ZDS1

ZIP1

ZMS1

ZPR1

Cluster13(node25)

Thereare319elements.

ACE2

ADK2

APA2

APM1

APM2

ARC15

ARP8

AXL1

BCK2

BDF1

BDP1

BOP1

BRO1

BZZ1

CAF40

CAM1

CAR1

CCC1

CCW12

CDC3

CFT2

CIN5

CIN8

CIT2

CMK2

CNE1

COX5A

COX9

CPR3

CSE4

CUP1-1

CUP1-2

CWC21

CYC3

CYC8

CYT1

DAL2

DAL5

DAL7

DAM1

DEP1

DPL1

DYN1

EDC1

EDC2

ENT3

ERG10

ERG12

ERS1

EXO1

FPS1

FUR4

GAT1

GAT2

GCR2

GFD1

GIM4

GLE1

GLG1

GPA2

GPB2

GYP5

HAP2

HAP5

HEM3

HFM1

HHT2

HIS3

HMG1

HXT7

ICY2

IES1

IMH1

INO2

IPT1

ISU1

IVY1

JIP4

JNM1

KEX2

KIP2

KRE11

LAG1

LEU5

LPE10

MAL11

MAL31

MBP1

MCM1

MCM10

MDH1

MDH2

MDM30

MEC3

MED2

MED7

MFT1

MIG1

MMS2

MRPL4

MSH5

MSH6

MSL5

MSS18

NCE2

NDJ1

NEJ1

NIT2

NKP1

NKP2

NTC20

OPT2

ORC4

OSH1

OSM1

PCL6

PET127

PEX22

PHD1

PHO87

PIL1

PIS1

PLB2

PMA2

PMT6

PPH21

PPZ2

PRM10

PRP28

PTK1

PXL1

QCR10

QCR2

QRI5

QRI7

RCS1

REC107

RED1

REV3

RFC3

RIP1

RLF2

RME1

RNP1

RNT1

ROX1

RRN3

RRN7

RRN9

RTS3

RVS161

SAC2

SDL1

SDS3

SEN15

SET5

SFP1

SGF73

SIF2

SKY1

SLG1

SLI15

SLU7

SLX8

SMF2

SNF11

SNF12

SNF5

SOD1

SOY1

SPE3

SPO16

SPS4

SPT10

SPT14

SPT16

SPT21

SRB5

SRV2

STP2

STP22

STU2

SUR1

SUT1

SWD2

SWH1

TAF8

TEL1

TEL2

TFA1

TIM22

TNA1

TOF2

TPC1

TPM2

TYE7

UBC6

UGA2

USO1

UTH1

UTR5

VAM7

VPS17

VPS28

YAP1802

YAR028W

YAR068W

YBL086C

YBR190W

YBR224W

YBR270C

YBR273C

YBR292C

YBR300C

YCK1

YCL065W

YCL068C

YCP4

YDL023C

YDL186W

YDR042C

YDR053W

YDR157W

YDR193W

YDR274C

YDR278C

YDR288W

YEL008W

YEL033W

YER134C

YGL108C

YGL109W

YGL193C

YGL214W

YGR004W

YGR052W

YGR071C

YGR102C

YGR107W

YGR137W

YGR242W

YGR243W

YHC1

YHR009C

YHR131C

YHR168W

YHR192W

YIL042C

YIL059C

YIL090W

YIP4

YIR016W

YIR036C

YJL086C

YJL107C

YJL131C

YJR013W

YJR083C

YJR085C

YJR087W

YJR088C

SFK1

YKL088W

YKL105C

YKR089C

YKR100C

YLR012C

YLR122C

YLR152C

YLR162W

YLR193C

YLR297W

YLR402W

YLR404W

YLR416C

YML036W

YML045W

YML090W

YMR051C

YMR088C

YMR102C

YMR134W

YMR278W

YMR294W-A

YMR295C

YNG2

YNL028W

YNL035C

YNL144C

YNL176C

YNL295W

YNR004W

YOL014W

YOL029C

YOL050C

YOL111C

YOR013W

YOR066W

YOR084W

YOR139C

YOR179C

YOR215C

YOR291W

YOR392W

YPL014W

YPL095C

YPL229W

YPR013C

YPR015C

YPR027C

YPR045C

YPR064W

YPR071W

YPR084W

YPR100W

YPR118W

YSA1

YSH1

ZEO1

Cluster14(node27)

Thereare131elements.

APG2

ARE2

ARR3

ATX2

BTN2

CAR2

CDC26

COQ2

COX5B

CSR2

CTF19

CTR3

DDR2

DOC1

ECM9

ELC1

FIT2

FKS3

FYV1

FYV2

GAC1

GIS3

GLO4

GSY2

HRR25

HSP104

HUR1

HVG1

ISF1

ISM1

KNH1

LSB1

MEP3

MRL1

MRP20

NVJ1

PAC1

PCD1

PDH1

PEX2

PEX6

PLB3

POG1

PRP18

PSY2

RAD59

RCO1

RMD5

SCC4

SET6

SFT1

SNA3

SPC42

TAF11

TES1

TIR2

TIR4

UIP4

VPS24

VRP1

WHI5

WSC4

YAL018C

YAL037W

YAR060C

YAT2

YBL029C-A

YBL095W

YBL096C

YCR079W

YDL109C

YDL110C

YDL242W

YDR070C

YDR095C

YDR391C

YDR493W

YEL028W

YGL146C

YGL199C

YGL211W

YGR146C

YGR149W

YGR268C

YGR269W

YHL010C

YHR198C

YIL057C

YIL060W

YIL161W

YIL167W

YIM2

YIP5

YIR020C

YJL007C

YJL028W

YJL144W

YJR020W

YJR056C

YJR100C

YJR115W

YJR120W

YJR129C

YKL023W

YKL086W

YKL202W

YLR168C

YLR343W

YLR349W

YLR350W

YML012C-A

YMR069W

YMR114C

YNL017C

YNL024C

YNL034W

YNL143C

YOL114C

YOR041C

YOR214C

YPK2

YPL056C

YPL114W

YPL166W

YPL185W

YPL230W

YPR090W

YPR093C

YPR127W

YPR157W

YTA6

Cluster15(node29)

Thereare105elements.

ALF1

AQY1

ARC19

ARF3

BMH1

BRR1

CCE1

CIT3

COX18

DIA4

DIC1

DRT1

DSK2

FCY21

FLO10

FLO5

FYV8

GAL4

GUT1

HEF3

HSE1

INP52

KIC1

KIP1

LSM2

MBR1

MIG3

MLH3

MOB2

MRPL27

MRPL40

MRPL9

MSO1

MTG1

MUD1

MUP1

NDD1

NDT80

NFT1

PET122

PHB1

QUT1

RFM1

RIB2

RIF2

RPN10

RTG1

RXT1

SBE2

SCD5

SCD6

SEC72

SLX4

SPC25

SPP1

SPP2

SRL3

SSN3

TFB3

THI22

TIP41

TOF1

UBP8

URA10

VTI1

YAP3

YBL060W

YBR134W

YBR269C

YCR045C

YCR076C

YDR360W

THI74

YGL085W

YGL138C

YGL247W

YGR042W

YGR153W

YGR235C

YHR199C

YHR213W

YJR114W

YKL069W

YKL075C

YKR043C

YLR030W

YLR072W

YLR077W

YLR446W

YML107C

YMR148W

YMR289W

YNL187W

YOR006C

YOR129C

YOR238W

YOR263C

YOR280C

YOR305W

YOR314W

YOR353C

YPL071C

YPL098C

YPR038W

YPR059C

Cluster16(node30)

Thereare56elements.

AQR1

ARP1

BUD31

CDD1

ECM13

FCP1

GOS1

HXT1

IDH1

IDH2

IFH1

INM1

IPI1

KAR5

KHS1

KRE34

LAS17

MAK21

NMD5

PAC11

PDC1

PEX21

RGS2

RPL39

RTS2

SIC1

SNC2

SNP1

SNU66

SPO19

SUV3

TAD3

TAT2

UBP10

YCR064C

YDL033C

YEL048C

YGL226W

YGR018C

YGR219W

YHL018W

YHR003C

YHR182C-A

YHR214W

YIL169C

YJU2

YKL076C

YLL012W

YLR211C

YLR400W

YML082W

YML087C

YOL155C

YPL144W

YPL182C

YPL206C
